# A distinct core regulatory module enforces oncogene expression in KMT2A-rearranged leukemia

**DOI:** 10.1101/gad.349284.121

**Published:** 2022-03-01

**Authors:** Taku Harada, Yaser Heshmati, Jérémie Kalfon, Monika W. Perez, Juliana Xavier Ferrucio, Jazmin Ewers, Benjamin Hubbell Engler, Andrew Kossenkov, Jana M. Ellegast, Joanna S. Yi, Allyson Bowker, Qian Zhu, Kenneth Eagle, Tianxin Liu, Yan Kai, Joshua M. Dempster, Guillaume Kugener, Jayamanna Wickramasinghe, Zachary T. Herbert, Charles H. Li, Jošt Vrabič Koren, David M. Weinstock, Vikram R. Paralkar, Behnam Nabet, Charles Y. Lin, Neekesh V. Dharia, Kimberly Stegmaier, Stuart H. Orkin, Maxim Pimkin

**Affiliations:** 1 Cancer and Blood Disorders Center, Dana-Farber Cancer Institute and Boston Children's Hospital, Harvard Medical School, Boston, Massachusetts 02215, USA;; 2 Broad Institute of Massachusetts Institute of Technology and Harvard, Cambridge, Massachusetts 02142, USA;; 3 The Wistar Institute, Philadelphia, Pennsylvania 19104, USA;; 4 Baylor College of Medicine, Houston, Texas 77030, USA;; 5 Ken Eagle Consulting, Houston, Texas 77494, USA;; 6 Dana-Farber Cancer Institute, Harvard Medical School, Boston, Massachusetts 02215, USA;; 7 Whitehead Institute for Biomedical Research, Cambridge, Massachusetts 02142, USA;; 8 Department of Biology, Massachusetts Institute of Technology, Cambridge, Massachusetts 02142, USA;; 9 Division of Hematology/Oncology, Department of Medicine, Perelman School of Medicine at the University of Pennsylvania, Philadelphia, Pennsylvania 19104, USA;; 10 Human Biology Division, Fred Hutchinson Cancer Research Center, Seattle, Washington 98109, USA;; 11 Howard Hughes Medical Institute, Boston, Massachusetts 02215, USA

**Keywords:** IRF8, KMT2A-rearranged AML, MEF2D, transcriptional addiction

## Abstract

Acute myeloid leukemia with KMT2A (MLL) rearrangements is characterized by specific patterns of gene expression and enhancer architecture, implying unique core transcriptional regulatory circuitry. Here, we identified the transcription factors MEF2D and IRF8 as selective transcriptional dependencies of KMT2A-rearranged AML, where MEF2D displays partially redundant functions with its paralog, MEF2C. Rapid transcription factor degradation followed by measurements of genome-wide transcription rates and superresolution microscopy revealed that MEF2D and IRF8 form a distinct core regulatory module with a narrow direct transcriptional program that includes activation of the key oncogenes MYC, HOXA9, and BCL2. Our study illustrates a mechanism of context-specific transcriptional addiction whereby a specific AML subclass depends on a highly specialized core regulatory module to directly enforce expression of common leukemia oncogenes.

## Abstract (precis)

In this study, Harada et al. identified the transcription factors MEF2D and IRF8 as selective transcriptional dependencies of KMT2A-rearranged AML, where MEF2D displays partially redundant functions with its paralog, MEF2C. This study illustrates a mechanism of context-specific transcriptional addiction whereby a specific AML subclass depends on a highly specialized core regulatory module to directly enforce expression of common leukemia oncogenes.

Metazoan development depends on cell type-specific gene expression programs established and reinforced by combinatorial actions of spatiotemporally restricted transcription factors (TFs) (Kerényi and Orkin 2010; Novershtern et al. 2011; Neph et al. 2012; Xu et al. 2012). Although an average mammalian cell expresses several hundred TFs, only a small number, variably referred to as reprogramming, master, or core regulatory TFs, are principally important for lineage specification (Davis et al. 1987; Boyer et al. 2005; Takahashi and Yamanaka 2006; Graf and Enver 2009; Orkin and Hochedlinger 2011). Core regulatory TFs form a network of interconnected feed-forward loops termed core regulatory circuitry (CRC) (Boyer et al. 2005; Saint-André et al. 2016). CRCs cooperatively enforce expression of key lineage genes by establishing extended, closely spaced enhancers with markedly high levels of histone acetylation and cofactor recruitment, termed superenhancers (Hnisz et al. 2013, 2015; Whyte et al. 2013). Superenhancer (SE) patterns are specific markers of cell identity and have been exploited to infer developmental and oncogenic programs, as well as therapeutic vulnerabilities (Chapuy et al. 2013; Lin et al. 2016; Mack et al. 2017; McKeown et al. 2017; Sengupta and George 2017; Mack et al. 2019).

Progression of a normal cell to a neoplastic state is associated with transcriptional derangement (Bradner et al. 2017). In addition to mutations in lineage TFs and chromatin regulators acting as common drivers of malignancy (Martínez-Jiménez et al. 2020), cancer cells may be critically dependent on nonmutated TFs enforcing cancer-specific transcriptional programs in a process referred to as transcriptional addiction (Zuber et al. 2011; Bradner et al. 2017; Lu et al. 2018; Tarumoto et al. 2018; Takao et al. 2021). For example, KMT2Ar leukemia is addicted to the transcription factor ZFP64 for the expression of its KMT2A fusion oncoprotein due to a naturally occurring high density of the ZFP64 motifs in the KMT2A promoter (Lu et al. 2018), while a somatic mutation creates a de novo MYB binding site driving overexpression of the TAL1 oncogene in T-cell acute lymphoblastic leukemia (Mansour et al. 2014). The transcriptional circuitry of a malignant cell thus includes elements retained from its cell of origin as well as cancer-specific circuits established de novo or subverted from the transcriptional programs of other developmental stages or lineages (Corces et al. 2016; Chen et al. 2020). In principle, these context-specific circuits may be integrated with the rest of a cell's core regulatory circuitry or represent distinct functional modules. In any case, to the extent that these de novo circuits are cancer-restricted, they offer new opportunities for targeted therapy (Bradner et al. 2017; Bushweller 2019; Henley and Koehler 2021).

Acute leukemias carrying KMT2A (MLL) translocations represent ∼5%–10% of acute leukemia in all age groups and up to 70% of infantile leukemia (Krivtsov and Armstrong 2007; Balgobind et al. 2009; Bolouri et al. 2018; Chan and Chen 2019). KMT2A fusion proteins drive leukemogenesis by recruiting the superelongation complex (SEC), Menin (MEN1), and the histone H3K79 methyltransferase DOT1L to drive overexpression of key leukemogenic transcription factors, such as HOXA9, MEF2C, and MEIS1 (Okada et al. 2005; Krivtsov et al. 2006, 2008, 2017; Krivtsov and Armstrong 2007; Wong et al. 2007; Faber et al. 2009; Bernt et al. 2011). Several features distinguish KMT2Ar leukemia as a malignancy associated with a deep rewiring of the normal transcriptional circuitry (Chan and Chen 2019). First, it is characterized by aberrant expression of multiple TFs and chromatin regulators (Armstrong et al. 2002; Ross et al. 2004; Vega-García et al. 2018). Second, it displays promiscuous expression of lineage markers and a propensity for lineage switching (Gagnon et al. 1989; Hudson et al. 1991; Cuneo et al. 1992; Stasik et al. 2006). Finally, it has been reported to be the only subtype of AML where the underlying genetic lesion is uniquely associated with a specific superenhancer profile (McKeown et al. 2017).

Here, we used an integrative functional genomics approach to characterize the transcription factors IRF8 and MEF2D as a selective transcriptional addiction of KMT2A-rearranged AML. Using rapid targeted protein degradation coupled with nascent transcriptomics and superresolution microscopy, we demonstrate that IRF8 and MEF2D form a KMT2Ar leukemia-specific core regulatory module directly enforcing expression of the common leukemia oncogenes MYC, HOXA9, and BCL2, where MEF2D displays partial functional redundancy with its paralog, MEF2C.

## Results

### A distinct transcriptional dependency profile of KMT2Ar AML

**Figure 1.**
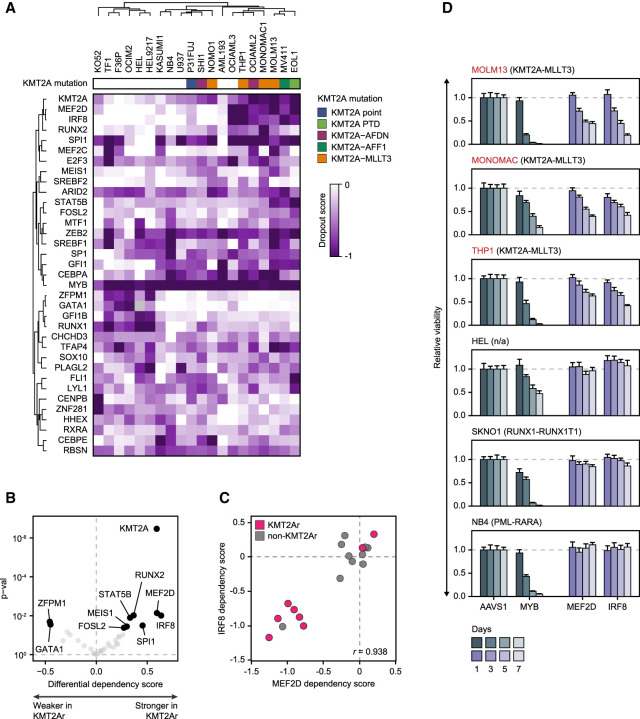
A distinct transcriptional dependency profile of KMT2Ar AML. (*A*) A heat map of CRISPR dropout scores of 35 selective transcriptional AML dependencies clustered by Pearson correlation with complete linkage, demonstrating a distinct dependency pattern shared by the majority of KMT2Ar cell lines. (*B*) A volcano plot of differential average dependency scores of the 35 selective transcriptional AML dependencies between KMT2Ar and non-KMT2Ar cell lines. Darker color corresponds to *P*-value < 0.05. (*C*) Correlation between IRF8 and MEF2D dependency scores in AML cell lines. Negative scores reflect stronger dependency. (*D*) Validation of MEF2D and IRF8 as selective dependencies of KMT2Ar leukemia using three cell lines carrying a KMT2A translocation versus three non-KMT2Ar cell lines. The cells were electroporated with in vitro assembled Cas9/sgRNA complexes targeting the indicated TF genes, and cell viability was measured relative to an AAVS1 (“safe harbor”) control by quantification of ATP pools using a luciferin-based assay. Knockout efficiency was confirmed by Western blot. MYB targeting sgRNAs were used as a positive control.

We reasoned that the relevant AML transcriptional circuitry could be most specifically identified by asking which TFs are selectively required for AML growth and survival (Durbin et al. 2018). We accessed the data from the Broad Cancer Dependency Map project, a collection of genome-scale CRISPR–Cas9 loss-of-function screens of 18,333 genes in 769 cell lines, including 20 AML lines (Meyers et al. 2017). Using a skewed LRT test (McDonald et al. 2017), we compared guide RNA dropout between AML and all other cell lines, allowing us to distinguish selective AML gene dependencies from universally essential housekeeping genes. We identified 225 genes that were selectively essential for the growth of AML cells, including 35 genes encoding TFs (Fig. 1A; Supplemental Fig. S1). KMT2Ar cell lines were characterized by a distinct dependency pattern, with stronger dependency scores for seven TFs (IRF8, MEF2D, SPI1, RUNX2, STAT5B, MEIS1, and FOSL2), in addition to the expected selective dependency on the KMT2A fusion. Among the seven KMT2Ar-specific TFs, IRF8 and MEF2D displayed the strongest selectivity for KMT2Ar AML, and their dependency scores displayed a high degree of correlation, suggesting a functional link (Fig. 1B,C). We independently confirmed the selective dependency of KMT2Ar leukemia on IRF8 and MEF2D by measuring cell viability after CRISPR–Cas9 TF knockout in a panel of six cell lines. Consistent with the results of the genome-scale screen, depletion of either IRF8 or MEF2D selectively inhibited growth of the cell lines carrying KMT2A translocations (Fig. 1D). Notably, high expression of IRF8 and MEF2D was associated with adverse outcomes in the TCGA AML data set, although the same effect did not reach statistical significance in the BeatAML data set (Supplemental Fig. S1; The Cancer Genome Atlas Research Network 2013; Tyner et al. 2018).

### Chromatin profiling reveals a divergent CRC structure in KMT2Ar AML

**Figure 2.**
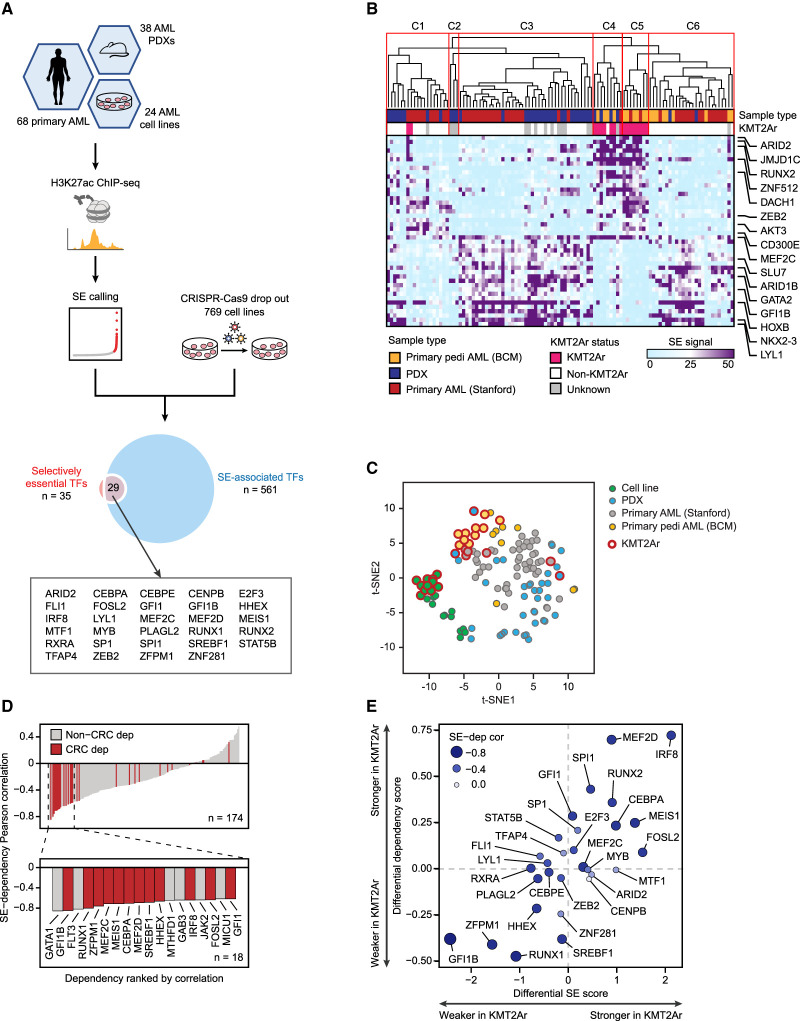
Divergent superenhancer landscapes are diagnostic of KMT2Ar-specific transcriptional vulnerabilities. (*A*) A study schematic depicting an integrative analysis of superenhancer landscapes and selective transcriptional dependencies to define the AML core regulatory circuitry (CRC). (*B*) Primary and PDX samples are hierarchically clustered using Pearson correlation of the scores of 4798 superenhancers recurrent in at least two samples. Superenhancers with the largest average score difference between KMT2Ar and non-KMT2Ar leukemias are selectively shown. (*C*) Samples of all types are plotted according to the superenhancer scores (4798 superenhancers recurrent in at least two samples) using t-distributed stochastic neighbor embedding (t-SNE). (*D*) Superenhancer-associated selective AML dependencies are ranked according to Pearson correlation between superenhancer scores and dependency. Core regulatory TFs are highlighted in red. (*E*) A differential plot of average superenhancer scores versus average dependency scores in KMT2Ar versus non-KMT2Ar cell lines.

We hypothesized that the selective dependency of KMT2Ar AML cell lines on a small subset of lineage TFs was due to a hierarchical CRC structure with distinct shared myeloid and KMT2Ar subtype-specific transcriptional circuits. As core regulatory TFs are typically associated with superenhancers (Saint-André et al. 2016; Chen et al. 2020), we sought to further define the common and divergent AML CRCs by integrating the transcriptional AML dependencies with the superenhancer structure (Fig. 2A). We assembled a mixed sample cohort with a strong representation of both adult and pediatric KMT2Ar AML, consisting of AML cell lines (*n* = 24), patient-derived xenografts (PDXs; *n* = 38) (Murakami et al. 2015), and pediatric primary AMLs (*n* = 19) (Perez et al. 2021). Additionally, we incorporated a published adult primary AML data set (*n* = 49) (McKeown et al. 2017), for a total of 130 samples. We performed chromatin immunoprecipitation sequencing (ChIP-seq) for the enhancer histone mark H3K27ac and identified a total of 6868 distinct superenhancers, of which 4798 were recurrent in at least two samples. In agreement with a prior report (McKeown et al. 2017), unsupervised clustering of leukemias by superenhancer structure significantly segregated KMT2A-rearranged samples into two related clusters (Fig. 2B; Supplemental Figs. S2, S3). A similar pattern was observed in cell lines (Fig. 2C; Supplemental Fig. S4), confirming unique and reproducible differences in the transcriptional circuitry imparted by the KMT2A translocations. On average, KMT2A-rearranged AMLs displayed a slightly lower number of superenhancers per sample compared with other AMLs (730 vs. 840, *P* = 0.028). To define the AML CRC, we intersected the 35 selectively essential TFs with 561 TF genes that were associated with recurrent AML superenhancers. The resulting list of 29 core TFs included all seven of the KMT2Ar-selective transcriptional dependencies (Fig. 2A). The degree of H3K27 acetylation within the TF-associated superenhancers strongly correlated with the TF dependency scores (Fig. 2D). Accordingly, KMT2Ar leukemias displayed significantly higher superenhancer scores associated with genes encoding KMT2Ar-selective transcriptional dependencies (Fig. 2E).

**Figure 3.**
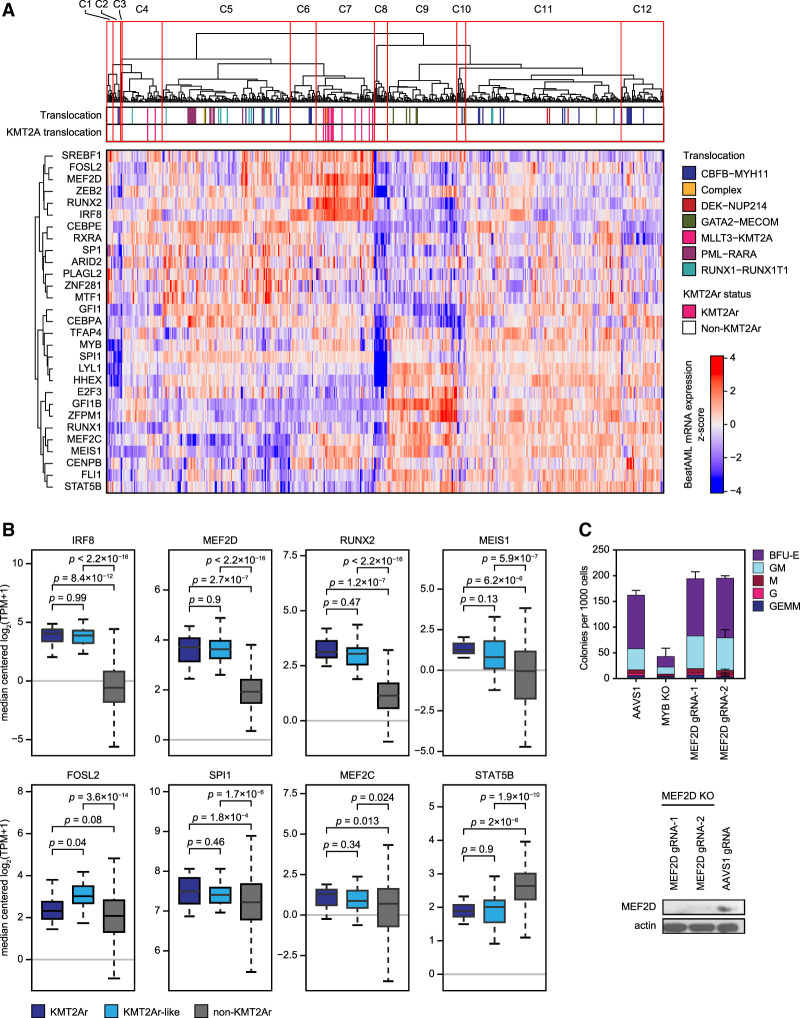
High expression of MEF2D and IRF8 marks a distinct cluster of KMT2Ar-like leukemia. (*A*) Classification of primary AML samples based on mRNA expression of core regulatory TFs. Samples from the BeatAML data set (*n* = 510) (Tyner et al. 2018) were hierarchically clustered using Pearson correlation of mRNA expression of the 29 core regulatory TFs with complete linkage. The heat map visualizes the *z*-scores of mRNA expression across the sample set. (*B*) Expression of the seven core regulatory TFs with stronger dependency scores in KMT2Ar cell lines, as well as MEF2C, in BeatAML samples. KMT2Ar-like samples are defined as samples coclustering with KMT2Ar leukemias (cluster 7) but not carrying a KMT2A translocation. (*C*) Human bone marrow-derived CD34^+^ cells from healthy donors were electroporated with in vitro assembled Cas9/sgRNA complexes targeting MYB and MEF2D and plated on cytokine-supplemented methylcellulose media. Colonies were counted following a 14-d incubation period. An AAVS1 (“safe harbor”) targeting sgRNA was used as a control. An efficient knockout of MEF2D in primary CD34^+^ cells was confirmed by Western blot, as shown.

To further validate the specific CRC signature of KMT2Ar leukemia, we analyzed patterns of TF expression in BeatAML, a large data set of 510 genetically annotated and mRNA-sequenced primary AMLs (Tyner et al. 2018). Hierarchical clustering of BeatAML samples by pairwise correlation of the 29 core TFs’ mRNA expression values revealed 12 major clusters. Leukemias carrying KMT2A rearrangements formed a single cluster, cosegregating with many leukemias having no major chromosomal rearrangements, which we termed KMT2Ar-like (Fig. 3A). Of the seven TFs characterized by stronger dependency in KMT2Ar cell lines, five TFs were significantly overexpressed in KMT2Ar and KMT2Ar-like AML (Fig. 3B). Two of these TFs (RUNX2 and MEIS1) are canonical transcriptional targets of the KMT2A fusion oncoproteins (Guenther et al. 2008; Bernt et al. 2011; Borkin et al. 2015; Krivtsov et al. 2019). Thus, the core TF expression signature of KMT2Ar leukemia in this independent patient cohort largely matched the superenhancer and gene dependency signatures of KMT2Ar AML. The KMT2A-like status was associated with a higher frequency of *FLT3* mutations but had no significant effect on patient survival in the BeatAML data set (Supplemental Fig. S5).

Because IRF8 and MEF2D were the most strongly selective transcriptional dependencies of KMT2Ar leukemia and most strongly overexpressed core TFs in primary KMT2Ar AML samples, we focused attention on these two TFs. Unlike IRF8, which plays an essential role in myeloid development (Tamura et al. 2015; Kurotaki et al. 2018), MEF2D has no assigned role in normal hematopoiesis, prompting us to hypothesize that it represents a leukemia-specific transcriptional addiction. Indeed, knockout of MEF2D in human CD34^+^ cells had no effect on myeloid colony formation (Fig. 3C), consistent with the published observation of normal hematopoiesis in *Mef2d*-null mice (Kim et al. 2008). In contrast, depletion of the master myeloid TF MYB resulted in a near-complete loss of colonies. While colony-forming assays are crude models of normal hematopoiesis, our results are consistent with MEF2D being a specific transcriptional addiction of KMT2Ar leukemia. In summary, our findings indicate that the divergent superenhancer patterns correspond to selective genetic vulnerabilities of KMT2Ar leukemia and reflect a functionally significant divergence of CRC structure in AML.

### Synthetic lethality reveals redundancy of MEF2 paralogs

**Figure 4.**
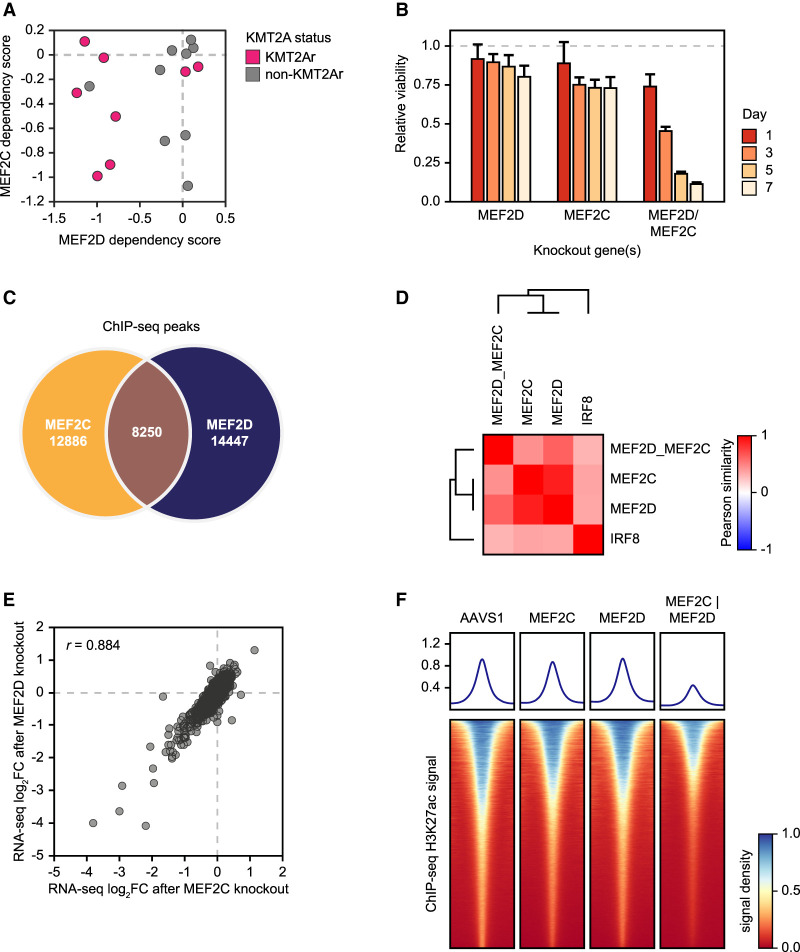
Functional redundancy of MEF2 paralogs. (*A*) A scatter plot of MEF2D/MEF2C dependency scores in AML cell lines. Negative scores correspond to stronger dependency. (*B*) Synthetic lethality of MEF2 paralogs. MV411 cells were electroporated with in vitro assembled Cas9/sgRNA complexes targeting one or both MEF2 paralogs as shown, and cell viability was measured relative to an AAVS1 (“safe harbor”) control by quantification of ATP pools using a luciferin-based assay. (*C*) Intersection of ChIP-seq peaks between MEF2D and MEF2C in MV411 cells. (*D*) A similarity matrix of TF knockouts hierarchically clustered by Pearson correlation between knockout-induced changes in the expression of the top 5000 expressed genes compared with the AAVS1 control. MV411 cells were electroporated with in vitro assembled Cas9/sgRNA complexes targeting the individual TFs as shown, as well as a simultaneous knockout of MEF2D and MEF2C, followed by RNA-seq. An AAVS1 (“safe harbor”) targeting sgRNA was used as a control. (*E*) Synergistic actions of MEF2D and MEF2C are illustrated by cross-plotting transcriptional responses of the top 5000 expressed genes to the MEF2D and MEF2C knockouts. (*F*) Changes in the genome-wide H3K27ac levels after designated TF knockouts, measured by quantitative ChIP-seq using an external spike-in control. Density plots depict genome-wide histone acetylation after the indicated TF knockouts. Each row visualizes spike-in-normalized ChIP-seq signal around a single H3K27ac peak.

The MEF2D paralog MEF2C is an established transcriptional addiction of KMT2Ar AML (Krivtsov et al. 2006; Brown et al. 2018; Tarumoto et al. 2018, 2019). However, dependency on MEF2C appears to have no significant selectivity toward KMT2Ar cell lines (Figs. 2E, 4A), and MEF2C is not as strongly overexpressed in KMT2Ar leukemias in the BeatAML data set (Fig. 3B). At the same time, while dependency on MEF2D is more specifically associated with KMT2A translocations, two KMT2Ar cell lines (SHI1 and NOMO1) are not dependent on either MEF2 paralog (Fig. 4A). We hypothesized that MEF2D and MEF2C were functionally redundant, allowing them to cross-compensate for each other's loss. Indeed, a simultaneous knockout of MEF2C and MEF2D was synthetically lethal in a cell line (MV411) that displayed only moderate growth inhibition when either paralog was depleted (Fig. 4B). To investigate further the functional relationship between MEF2D and MEF2C, we first examined their genomic binding preference by chromatin immunoprecipitation sequencing (ChIP-seq), which revealed partially overlapping patterns of chromatin occupancy (Fig. 4C). Next, we examined the functional consequences of inactivating MEF2D and MEF2C by CRISPR/Cas9 editing and measuring the transcriptional response by RNA-seq. Knockouts of the two paralogs produced highly concordant transcriptional responses, indicating a significant functional redundancy (Fig. 4D,E). A concomitant depletion of MEF2D and MEF2C resulted in a more profound genome-wide loss of H3K27 acetylation compared with depletion of either paralog alone, consistent with their synthetic lethality (Fig. 4F). In summary, our observations indicated both redundant and unique functions of the MEF2 paralogs in AML, where MEF2D displays a stronger specificity for KMT2Ar leukemia.

### The IRF8/MEF2 module regulates key oncogenes in KMT2Ar AML

**Figure 5.**
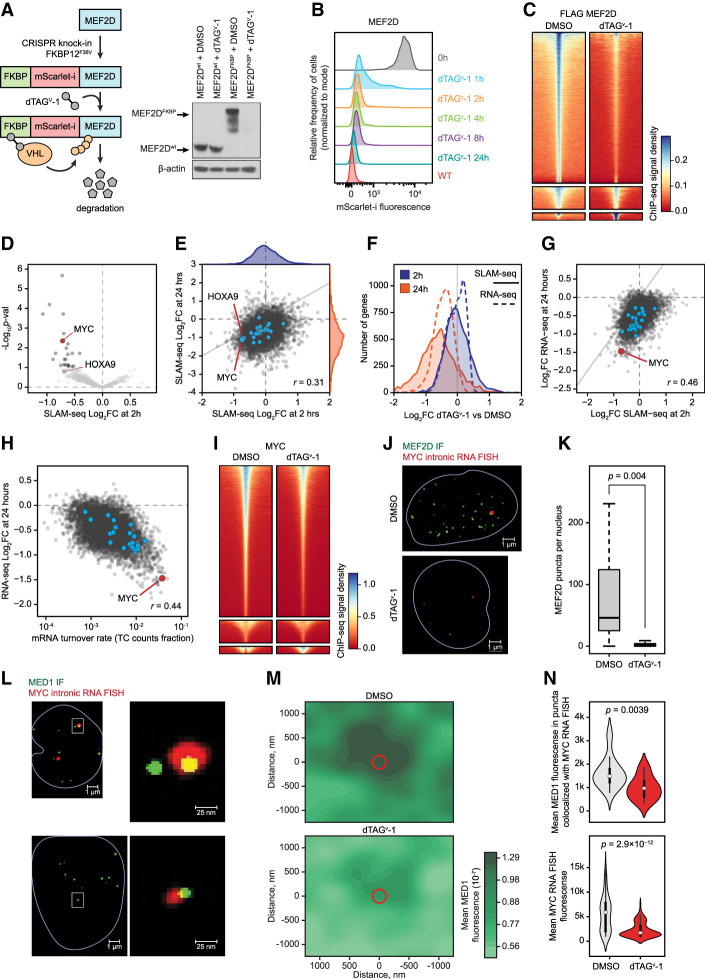
Direct transcriptional effects of MEF2D revealed by targeted degradation and SLAM-seq. (*A*) Schematic and Western blot of endogenous MEF2D tagging by CRISPR–HDR and subsequent targeted degradation of the fusion protein. (*B*) A time course of MEF2D degradation by FACS measurement of the fusion protein fluorescence. (*C*) Degradation of MEF2D reduces its genomic occupancy, as demonstrated by density plots of spike-in-controlled anti-Flag MEF2D ChIP-seq experiment showing genome-wide occupancy change after MEF2D degradation. Each row represents a single peak. (*D*) A volcano plot of genome-wide changes in nascent mRNA transcription measured by SLAM-seq after 2 h of MEF2D degradation. (*E*) A cross-plot of genome-wide changes in nascent mRNA transcription measured by SLAM-seq after 2 versus 24 h of MEF2D degradation demonstrates a poor correlation between early and late transcriptional responses, as well as signs of transcriptional collapse by 24 h. (*F*) A distribution plot of genome-wide changes in nascent transcription rates (SLAM-seq) and mRNA pools (RNA-seq) after 2 and 24 h of MEF2D degradation. (*G*) Correlation between changes in nascent RNA transcription (SLAM-seq) after 2 h of MEF2D degradation versus changes in the mRNA pools (RNA-seq) after 24 h of MEF2D degradation. (*H*) Correlation between steady-state mRNA turnover rates approximated from the SLAM-seq TC count fraction versus changes in the mRNA pools (RNA-seq) after 24 h of MEF2D degradation. (*I*) A density plot of spike-in-controlled anti-MYC ChIP-seq experiment demonstrating reduced genome-wide MYC occupancy after MEF2D degradation. (*J*) Degradation of MEF2D for 2 h results in a dramatic reduction of MEF2D puncta in the nucleus. (Green) MEF2D immunofluorescence (IF) signal, (red) intronic MYC RNA FISH signal. (*K*) Quantitative analysis of MEF2D puncta after degradation. (*L*) Degradation of MEF2D for 2 h results in decreased mediator recruitment and reduced MYC transcription. (Green) MED1 IF signal, (red) intronic MYC RNA FISH signal, (yellow) overlap between the red and green signals. (*M*) A density plot of multi-image analysis showing reduced mediator recruitment to foci of MYC transcription after degradation of MEF2D for 2 h. The green color gradient represents kernel density estimation of aggregate MED1 IF signal in a cubic region of 1400 nm^3^ centered on the MYC RNA FISH puncta in each cell. The red circle represents the average size of the MYC RNA FISH puncta. (*N*) Quantitative analysis of MED1 IF and MYC RNA FISH puncta before and after degradation demonstrating reduced mediator and RNA fluorescence at the sites of MYC transcription after MEF2D degradation for 2 h.

The tight linkage between the IRF8 and MEF2D dependencies prompted us to hypothesize that these TFs formed a distinct core regulatory module in KMT2Ar AML. To identify genes directly regulated by MEF2D and IRF8, we used a targeted protein degradation strategy (Muhar et al. 2018; Nabet et al. 2018, 2020). We constructed two AML cell lines expressing MEF2D and IRF8, respectively, fused to the FKBP12^F36V^ (dTAG) domain by a homozygous knock-in of the FKBP12^F36V^-coding DNA sequence into the endogenous TF-coding loci (Figs. 5A–C, 7A–C, below). Treatment with dTAG^V^-1, a heterobifunctional small molecule that engages FKBP12^F36V^ and VHL (Nabet et al. 2020), led to a near-complete loss of the fusion protein within 2 h. The effects of TF degradation on cell growth were quantitatively similar to the growth inhibition seen after CRISPR–Cas9-mediated gene knockouts (Supplemental Fig. S6). Following degradation of MEF2D and IRF8 for 2 and 24 h, we measured the genome-wide rates of nascent mRNA synthesis by SLAM-seq (Herzog et al. 2017).

Remarkably, degradation of MEF2D for 2 h resulted in a significant decrease in transcription of only 23 genes (adjusted *P*-value < 0.1) (Fig. 5D; Supplemental Figs. S7, S8), representing high-confidence direct MEF2D targets. Of these genes, seven encoded TFs (MYC, BHLHE40, KLF10, KLF6, KLF11, ZBTB33, and ZATB2) and two were essential genes (MYC and TP53RK) with dependency probability >0.5 (Supplemental Fig. S8). Two additional myeloid TFs (ZEB2 and HOXA9) had borderline statistical significance at 2 h but became significant by 24 h, likely also representing direct MEF2D targets. By 24 h of MEF2D deprivation, a global decrease in transcription was evident, consistent with a global transcriptional collapse (Fig. 5E–G). However, the changes in the transcription rates of individual genes seen after 24 h of MEF2D deprivation correlated poorly with the early changes, indicating that the majority of the late transcriptional responses were secondary events. Thus, although at least eight core regulatory TFs were significantly affected at 24 h, including IRF8 and MEF2D itself, these appear to be secondary effects rather than direct MEF2D targets. The global decrease in mRNA pools by 24 h, measured by RNA-seq, lagged behind the changes in transcription rates and correlated with mRNA turnover rates (Fig. 5H).

Given the role of MYC as a “transcriptional amplifier” (Lin et al. 2012), reduced MYC activity, evidenced by decreased MYC binding across the genome after MEF2D degradation (Fig. 5I), likely contributed to the global transcriptional collapse. Indeed, we found the MYC transcriptional signature to be strongly enriched among the genes affected by the MEF2D loss (Supplemental Fig. S9). Furthermore, indirect inhibition of MEF2 function via inhibition of SIK3 by the small molecule tool compound YKL-05-099 (Tarumoto et al. 2019) had a similar direct transcriptional signature, including reduced MYC transcription, and demonstrated a modest synergy with pharmacologic MYC inhibition (Supplemental Fig. S9). However, forced expression of MYC failed to rescue the growth phenotype of MEF2D degradation (Supplemental Fig. S9), consistent with the observation that direct MEF2D targets include other essential genes.

**Figure 6.**
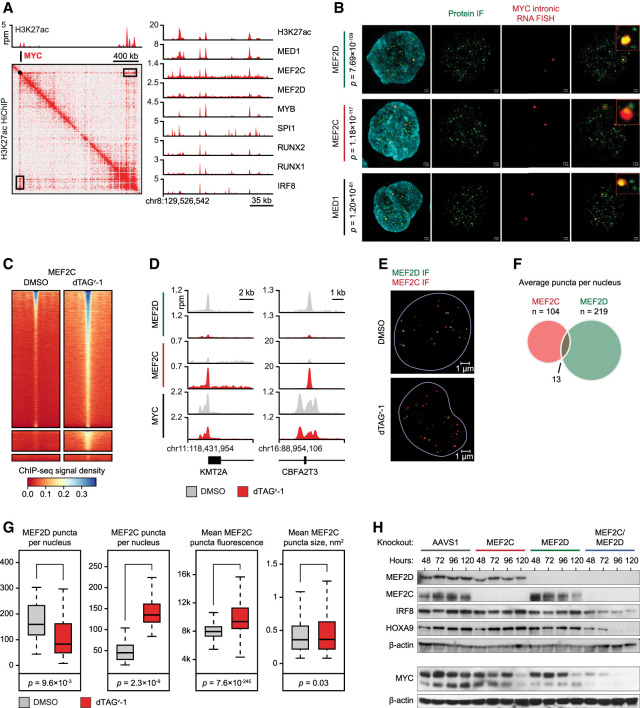
Compensation and competition between MEF2D and MEF2C. (*A*) A 2D HiChIP plot illustrating H3K27ac-mediated DNA contacts in the *MYC* locus and ChIP-seq tracks of core regulatory TF binding at the MYC SE located ∼1.7 Mb downstream from the MYC promoter (black box on the HiChIP map). (*B*) SIM superresolution confocal microscopy of MV411 cells with simultaneous immunofluorescence using primary antibodies against the designated proteins and intronic RNA FISH targeting nascent MYC transcripts. *P*-values reflect significance of colocalization of protein puncta with RNA FISH calculated by Fisher exact test. (*C*) A density plot of a spike-in-controlled anti-MEF2C ChIP-seq experiment demonstrating increased MEF2C occupancy after MEF2D degradation. Each row represents a single peak called from an anti-Flag MEF2D ChIP-seq experiment in unperturbed MV411 cells. Color gradient reflects MEF2C ChIP-seq signal registered in the MEF2D peaks. (*D*) ChIP-seq tracks demonstrating changes in MEF2D, MEF2C, and MYC binding in two representative loci after MEF2D degradation. (*E*) MEF2D degradation results in an increased number and intensity of MEF2C nuclear puncta. The images demonstrate immunofluorescence with antibodies against MEF2D (green) and MEF2C (red) before and after MEF2D degradation. (*F*) Overlap between MEF2D and MEF2C puncta in unperturbed cells on multi-image analysis. (*G*) Quantitative multi-image analyses of MEF2D and MEF2C puncta before and after MEF2D degradation shows increased number and intensity of MEF2C condensates after MEF2D degradation. (*H*) Western blot demonstrating changes in TF protein levels after single and combined MEF2D/MEF2C knockouts by CRISPR/Cas9.

We sought additional confirmation of a direct role of MEF2D in the regulation of MYC transcription. First, we interrogated the 2D chromatin structure and TF occupancy in the *MYC* locus. HiChIP analysis of DNA loops associated with the H3K27ac histone mark revealed a large superenhancer located ∼1.7 Mb downstream from the *MYC* TSS, making extensive contacts with the *MYC* locus (Fig. 6A). This superenhancer corresponds to the previously described enhancer cluster regulating normal and malignant hematopoiesis (Shi et al. 2013; Bahr et al. 2018). Indeed, ChIP-seq revealed dense cobinding of MEF2D with other core AML TFs in this region (Fig. 6A). Next, we asked whether MEF2D binding to the *MYC* locus promoted MYC expression by facilitating mediator recruitment. Using superresolution structured illumination microscopy (SIM) immunofluorescence (IF), we visualized MEF2D puncta in the nucleus. The MEF2D puncta significantly colocalized with the sites of active MYC transcription highlighted by concurrent fluorescent in situ hybridization (FISH) for nascent MYC RNA (Figs. 5J, 6B). Degradation of MEF2D resulted in reduced intensity of MED1 puncta, along with a reduced intensity of the nascent RNA FISH signal, consistent with impaired MYC transcription (Fig. 5J–N). These results confirmed that MEF2D directly activates MYC transcription by binding to the MYC superenhancer and promoting mediator recruitment.

We hypothesized that the modest immediate transcriptional response to MEF2D loss was due to compensation by MEF2C. Indeed, both paralogs localized to the MYC locus on SIM IF-FISH and ChIP-seq (Fig. 6A,B). Remarkably, degradation of MEF2D caused redistribution of MEF2C to the genomic sites vacated by MEF2D (Fig. 6C,D). Similarly, MEF2C nuclear puncta, which only partially overlapped with MEF2D puncta on SIM IF, increased in number and intensity following MEF2D degradation despite a stable or slightly decreased total MEF2C level (Fig. 6E–G; Supplemental Fig. S7). These results are consistent with a genome-wide competition between MEF2D and MEF2C for chromatin binding and provide a mechanistic basis for their partially redundant functions. Consistent with these observations, simultaneous knockouts of MEF2D and MEF2C resulted in a more profound loss of MYC and HOXA9 compared with knockouts of either paralog alone (Fig. 6H).

**Figure 7.**
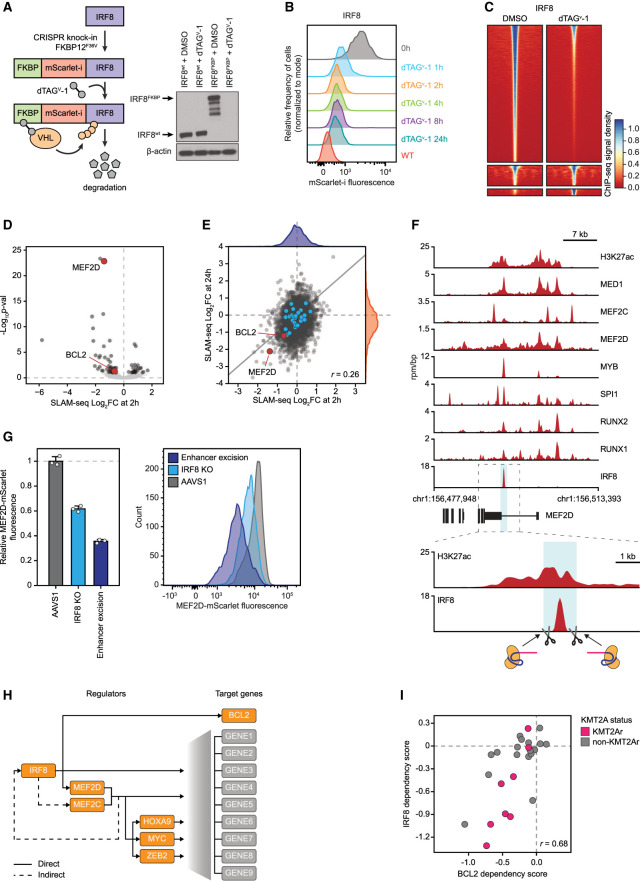
IRF8 directly regulates MEF2D and BCL2. (*A*) Schematic and Western blot of endogenous IRF8 tagging by CRISPR–HDR and subsequent targeted degradation of the fusion protein. (*B*) A time course of IRF8 degradation by FACS measurement of the fusion protein fluorescence. (*C*) A density plot of spike-in-controlled anti-IRF8 ChIP-seq experiment showing reduced genome-wide occupancy after degradation. (*D*) A volcano plot of genome-wide changes in nascent mRNA transcription measured by SLAM-seq after 2 h of IRF8 degradation. (*E*) A cross-plot of genome-wide changes in nascent mRNA transcription measured by SLAM-seq after 2 versus 24 h of IRF8 degradation demonstrates a poor correlation between early and late transcriptional response. (*F*) ChIP-seq tracks of core regulatory TF binding at the MEF2D superenhancer and schematic of CRISPR/Cas9 strategy for IRF8 binding site excision. (*G*) Changes in the MEF2D protein levels measured by mScarlet reporter fluorescence after IRF8 gene knockout versus excision of the IRF8 binding site in the MEF2D locus. MV411 cells carrying the FKBP-mScarlet-MEF2D fusion were electroporated with Cas9/sgRNA complexes targeting the IRF8 gene, IRF8 binding site in the MEF2D SE, or AAVS1 control, respectively, and fluorescence was measured by FACS 72 h after electroporation. (*H*) Schematic of the direct and indirect regulatory relationships in the IRF8/MEF2 axis. (*I*) Correlation between BCL2 and IRF8 dependency scores in AML cell lines.

Targeted degradation of IRF8 for 2 h resulted in a significant loss of MEF2D transcription, providing an explanation for the functional linkage between the two TFs (Fig. 7A–E; Supplemental Fig. S10). Although MEF2D was the only affected TF, direct targets of IRF8 included 71 additional genes, of which six were essential, including the BCL2 oncogene (Supplemental Figs. S10, S11). We found a single strong IRF8 binding site in the MEF2D superenhancer (Fig. 7F), prompting us to hypothesize that IRF8 activated MEF2D transcription by binding at this locus. We excised an ∼340-bp DNA sequence containing the IRF8 binding motif in the cell line carrying the FKBP-mScarlet-MEF2D fusion and measured mScarlet fluorescence as a reporter for MEF2D expression. Loss of the IRF8 binding sequence resulted in reduced fluorescence, indicating that the superenhancer segment containing the IRF8 binding site is essential for maintaining MEF2D expression (Fig. 7G). Notably, concurrent depletion of MEF2D and MEF2C resulted in decreased IRF8 levels (Fig. 6H), while a prolonged depletion of either IRF8 or MEF2D reduced the levels of MEF2C (Supplemental Figs. S7, S10). However, the absence of similar changes on SLAM-seq after rapid TF degradation indicated that these connections were indirect.

### A developmental mechanism of IRF8/MEF2D axis activation

Since IRF8 is not among the canonical transcriptional targets of KMT2A fusion oncoproteins (Milne et al. 2002, 2005; Krivtsov et al. 2006, 2008, 2017; Krivtsov and Armstrong 2007; Guenther et al. 2008; Bernt et al. 2011; Kerry et al. 2017), we explored how IRF8 expression may be activated in KMT2A-rearranged AML. Among the TFs that we and others identified to be selectively essential and overexpressed in KMT2A-rearranged leukemia, MEF2C, RUNX2, and MEIS1 are well-established direct targets of KMT2A fusion oncoproteins (Krivtsov et al. 2006, 2019; Bernt et al. 2011), prompting us to consider that one of these TFs drives *IRF8* overexpression. The *IRF8* locus contains two superenhancers, both of which form extensive contacts with the IRF8 promoter on HiChIP and Micro-C analyses (Supplemental Fig. S12). Using the dCas9-KRAB-MeCP2 technology to directly inhibit enhancer activity (Yeo et al. 2018), we observed a particularly strong reduction of the IRF8 expression and cell growth with the inhibition of two enhancers located +79 and +83 kb from the TSS, respectively, both of which were co-occupied by MEF2C, RUNX2, and MEIS1 (Supplemental Fig. S12). However, inactivation of MEF2C by CRISPR did not alter IRF8 expression, while knockouts of RUNX2 and MEIS1 resulted in a modest increase of IRF8 expression (Supplemental Fig. S12). Thus, we concluded that KMT2A fusion oncoproteins do not activate the IRF8/MEF2D axis either directly or via the canonical KMT2A fusion-activated TFs. In agreement with our observations, pharmacologic inhibition of DOT1L and MEN1, two essential cofactors that interact with KMT2A oncoproteins and mediate leukemogenic gene expression, decreased expression levels of MEF2C, RUNX2, and MEIS1 but had no effect on the expression of IRF8 or MEF2D (Borkin et al. 2015; Kerry et al. 2017; Godfrey et al. 2019; Krivtsov et al. 2019). Since IRF8 is highly expressed in committed granulocyte–monocyte progenitors (GMPs), we hypothesized that IRF8 overexpression in KMT2A-rearranged AML may be programmed by its cellular differentiation state. Indeed, classifying AML samples according to their myeloid differentiation stage revealed that IRF8 expression strongly correlates with a more mature myeloid phenotype characteristic of KMT2A-rearranged AML (Supplemental Fig. S13).

In summary, we conclude that IRF8 and MEF2D are context-specific transcriptional addictions of KMT2A-rearranged AML, where IRF8 directly activates transcription of MEF2D, while direct targets of MEF2D include key leukemogenic TFs HOXA9 and MYC (Fig. 7H). Direct targets of both TFs include other essential genes, including IRF8 regulation of BCL2, an enriched dependency, and a strong therapeutic target in AML (Wei et al. 2020). Indeed, BCL2 and IRF8 dependency scores display a significant degree of correlation (Fig. 7I; Supplemental Fig. S1). Remarkably, both MEF2D and IRF8 directly regulate relatively small, nonoverlapping sets of target genes, and most of the transcriptional changes detected by RNA-seq after a TF knockout represent secondary effects.

## Discussion

Using an unbiased approach to screen for AML transcriptional addictions, we identified the transcription factors IRF8 and MEF2D as selective dependencies of KMT2Ar AML. Both TFs have been previously noted to be overexpressed in KMT2Ar leukemia and associated with adverse outcomes (Armstrong et al. 2002; Krivtsov et al. 2006; Vega-García et al. 2018). To elucidate the mechanistic basis of the KMT2Ar leukemia addiction to MEF2D and IRF8, we set out to establish their direct gene regulatory functions. Traditional gene or messenger RNA disruption methods followed by measurements of mRNA pools cannot distinguish primary TF targets from secondary effects due to the slow onset kinetics of TF deprivation and the vast differences in mRNA and protein turnover rates (Schwanhäusser et al. 2011; Housden et al. 2017; Mathieson et al. 2018). Recently, direct TF targets have been inferred from direct measurements of genome-wide transcription rates following rapid TF degradation (Muhar et al. 2018; Stengel et al. 2021). A potential limitation of this approach is that some bona fide direct TF targets may have a slower response to rapid TF degradation and will be missed by a fast kinetics experiment, while a slower approach would preclude the distinction between the direct and indirect TF targets. Nonetheless, leveraging this approach, we demonstrate that (1) IRF8 and MEF2D form a distinct core regulatory module with a very narrow direct transcriptional program in KMT2Ar leukemia; (2) the IRF8/MEF2D module directly enforces expression of the key leukemia oncogenes MYC, HOXA9, and BCL2; and (3) MEF2D has partially redundant functions with its paralog, MEF2C. We provide additional evidence of direct regulation of MYC expression by MEF2D by direct visualization of reduced mediator puncta at the MYC locus after MEF2D degradation. Intriguingly, while core regulatory TFs are typically expected to regulate their own and each other's expression, the IRF8/MEF2D axis demonstrates a unidirectional flow of direct regulation, where IRF8 regulates MEF2D but not vice versa, and neither TF shows evidence of self-regulation. Furthermore, with few exceptions, the IRF8/MEF2D module does not regulate other members of the AML CRC. In that regard, IRF8 and MEF2D may represent a highly specialized auxiliary transcriptional module rather than a fully integrated subcircuit within the CRC. While we did observe decreased IRF8 levels after a concomitant depletion of both MEF2D and MEF2C by CRISPR, elucidating whether this represents direct regulation of IRF8 by the redundant MEF2 paralogs versus an indirect effect would require a fast kinetics experiment with simultaneous degradation of both TFs.

Importantly, rather than regulating a KMT2Ar-specific transcriptional program, the IRF8/MEF2D module enforces expression of ubiquitous leukemia oncogenes. Indeed, MYC, HOXA9, and BCL2 have well-established roles in AML leukemogenesis and maintenance. MYC is one of the most prominent human proto-oncogenes and a well-established therapeutic target (Dang 2012; Bahr et al. 2018; Duffy et al. 2021; Henley and Koehler 2021). It is universally essential in all surveyed AML cell lines (Supplemental Data) and a strong predictor of AML survival and chemoresistance (Luo et al. 2005; Delgado et al. 2013; Ohanian et al. 2018; Carter et al. 2020). HOXA9 is a common driver of leukemia, one of the strongest predictors of survival in AML patients, and a transcriptional addiction in KMT2Ar AML (Andreeff et al. 2008; Faber et al. 2009; Dang 2012; Collins and Hess 2016). The antiapoptotic protein BCL2 is another AML oncogene, a predictor of AML survival and chemoresistance, and the target of venetoclax, one of the most efficacious novel therapies for AML/MDS in combination with azacytidine (Campos et al. 1993; Lagadinou et al. 2013; DiNardo et al. 2020; Wei et al. 2020). The observation that KMT2A-rearranged leukemias depend on the IRF8/MEF2D axis for expression of common leukemia oncogenes raises the question of how expression of these oncogenes is maintained in non-KMT2Ar contexts. Indeed, several of the IRF8/MEF2D direct target genes, such as MYC, HOXA9, and ZEB2, are highly expressed and essential in non-KMT2Ar leukemias and normal hematopoietic progenitors that do not express high levels of IRF8/MEF2D. Such convergence of context-specific regulatory pathways on shared (proto-)oncogenes is a common phenomenon, with MYC providing a well-studied example (Dang 2012).

Functional redundancy of coexpressed TF paralogs has been recognized as one of the mechanisms providing robustness to gene regulatory networks (Hollenhorst et al. 2007; MacNeil and Walhout 2011; Collins and Hess 2016). However, the mechanisms by which TF paralogs exert their similar but not identical functions are not well understood. Our study uncovers partially redundant functions of MEF2C and MEF2D in KMT2Ar AML. While MEF2C is a well-established transcriptional dependency of KMT2A-rearranged AML (Krivtsov et al. 2006; Brown et al. 2018; Tarumoto et al. 2018), the role of MEF2D in leukemia has remained relatively unexplored. Although MEF2C and MEF2D are characterized by partially divergent binding patterns consistent with distinct binding codes, MEF2C compensates for MEF2D loss by relocating to the MEF2D binding sites and displaying increased puncta in the nucleus. Consistent with our observations, MEF2A has been reported to attenuate loss of MEF2D in mouse cerebellum (Majidi et al. 2019). Only MEF2D appears to be directly regulated by IRF8. Indeed, MEF2D expression correlates with IRF8 expression in primary AML samples, while the expression of MEF2C is more widely distributed and correlates with the expression of HOXA9 in non-KMT2Ar AML (data not shown). Thus, MEF2C may activate expression of HOXA9 in non-KMT2Ar leukemias, while the role of MEF2D is restricted to KMT2Ar AML. Intriguingly, ectopic expression of MEF2C has been shown to induce leukemic transformation in *Irf8*^−/−^ mice (Schwieger et al. 2009). In light of our data, it appears that IRF8 knockout resulted in a loss of MEF2D expression, which was then functionally rescued by overexpression of MEF2C, promoting leukemogenesis. We suspect that paralog redundancy is a common feature of transcription networks and has important implications for the interpretation of gene dependency screens. For example, direct targets of MEF2D include three KLF paralogs (KLF10, KLF6, and KLF11). While none of the individual paralogs are essential in the genome-wide dependency screen, the simultaneous loss of all three proteins may be synthetically significant and contribute to the eventual transcriptional collapse triggered by the MEF2D degradation.

Several recent reports have explored the roles of IRF8 and MEF2D in AML (Cao et al. 2021; Liss et al. 2021; Zhao et al. 2021). In Zhao et al. (2021), MEF2D is reported to inhibit a CEBPE-mediated program of leukemia differentiation. While we observed increased expression of CEBPE after either IRF8, MEF2D, or combined MEF2D/MEF2C knockouts (Supplemental Data), our direct transcriptomics data indicate that CEBPE may not be a direct MEF2D target. Similarly, Cao et al. (2021) reported that the transcriptional regulator ZMYND8 activates expression of MYC and IRF8, and IRF8 and MEF2D enforce each other's expression. While we did observe decreased IRF8 levels after a prolonged depletion of MEF2D, our data suggest that the reciprocal regulation of IRF8 by MEF2D is either indirect or, as noted above, largely redundant with MEF2C. Notably, while ZMYND8 is an enriched AML dependency (Supplemental Fig. S1), it shows no selectivity toward KMT2Ar AML, indicating that it plays a more ubiquitous role.

While our data do not reveal a fusion protein-driven mechanism of IRF8/MEF2D axis activation in KMT2A-rearranged leukemia, we found that it correlates with the degree of myeloid differentiation. This relationship is a common feature of context-specific transcriptional addiction where TF dependency is often imposed by the developmentally programmed transcriptional state of the cancer cell of origin rather than being directly established by the tumor-initiating mutation(s) (Hnisz et al. 2015; Boeva et al. 2017; Bradner et al. 2017; Mack et al. 2017, 2019; van Groningen et al. 2017). Indeed, KMT2A-rearranged AML tends to have a maturing myeloid phenotype (M4 and M5 in the French–American–British classification) (Tien et al. 2000), and, in normal hematopoiesis, expression of IRF8 is highest in granulocyte–monocyte progenitors (Supplemental Fig. S13). Similarly, in mouse models, while both HSCs and committed myeloid progenitors can be transformed by KMT2A fusions, the resulting leukemia stem cells (LSCs) assume a differentiating myeloid progenitor phenotype (Krivtsov et al. 2006, 2013; Krivtsov and Armstrong 2007). Thus, activation of the IRF8/MEF2D axis largely reflects the myeloid trajectory of KMT2A-rearranged AML. This concept is further illustrated by the KMT2A-like leukemias, which express high levels of IRF8 and MEF2D despite the absence of a KMT2A translocation.

In summary, leveraging fast kinetics TF degradation and nascent transcriptomics, our study provides an example of a specific class of AML relying on a distinct module within the core transcriptional hierarchy to enforce expression of common, rather than context-specific, oncogenes. Further studies will be needed to elucidate the mechanistic basis of the IRF8/MEF2D module activation and the selective, context-specific dependence on these TFs for the expression of common oncogenes in KMT2Ar leukemia.

## Materials and methods

### Key resources

A list of key reagents, constructs and resources is in Supplemental Data 1.

### Experimental model and subject details

#### Cell lines and patient-derived xenograft samples

AML cell lines were cultured in the RPMI-1640 media containing 10% fetal bovine serum and regularly tested to be free of Mycoplasma spp. Patient-derived xenograft samples (PDXs) were obtained from the DFCI PRoXe repository (https://proxe.shinyapps.io/PRoXe). PDX cytogenetics and molecular characteristics were obtained from the PRoXe/cBioPortal database (Cerami et al. 2012; Gao et al. 2013; Murakami et al. 2015).

#### Primary AML samples

Samples from children with AML were obtained with informed consent, according to protocols approved by the Institutional Review Board of Baylor College of Medicine. Patients’ cytogenetics and molecular characteristics were collated from clinical chart records. At the time of collection, mononuclear cells were enriched by density centrifugation and cryogenically preserved. Samples selected for inclusion contained high purity of blasts (>85%). Cells were preserved in conditioned media from the human bone marrow stromal cell line HS5 (collected after 2 d of plating) diluted 1:1 with RPMI + 10% FBS and 1% penicillin/streptomycin. Freshly thawed cells were cross-linked with formaldehyde and used for ChIP-seq (Perez et al. 2021).

### Methods

#### CRISPR/Cas9 gene knockouts by RNP electroporation

Synthetic modified sgRNA constructs were purchased from Synthego. Ribonucleoprotein (RNP) assembly was performed by mixing two to three sgRNAs (a total of 120 pmol) with 8.5 µg of recombinant Cas9 (Invitrogen A36499). The resulting RNP mix was electroporated into 0.3 × 10^6^ MV411 cells using a Lonza 4D nucleofector, program DJ-100, in 20 µL of nucleocuvette strips (Lonza V4XC-2032). Unless otherwise noted, cells were incubated in media for 72 h after electroporation before subsequent analyses. Knockout efficiency was confirmed by Western blotting and PCR amplification followed by indel analysis. A guide RNA targeting the AAVS1 “safe harbor” locus was used as a negative control. Cell viability was measured at the indicated times after electroporation using the CellTiter-Glo luminescent cell viability assay (Promega G7570).

#### Chromatin immunoprecipitation sequencing (ChIP-seq)

ChIP-seq for hematopoietic TFs was performed using 100 × 10^6^ exponentially growing MV411 cells per experiment. For histone ChIP-seq, 2.5 × 10^6^ cells were used. Cells were fixed with 1% formaldehyde for 10 min at room temperature, quenched with 125 mM glycine for 5 min, and washed three times with PBS. Nuclei were isolated using Nuclei EZ isolation buffer (Sigma NUC-101) and resuspended in 10 mM Tris-HCl (pH 8.0), 1 mM EDTA, and 0.1% SDS with 1× HALT protease inhibitor (Thermo Fisher 78430). Chromatin was fragmented by sonication on an E220 Covaris focused sonication machine using 1-mL glass AFA tubes (Covaris 520135) with the following parameters: 140 mV, 5% duty factor, and 200 cycles/burst for 14 min. The rest of the ChIP-seq protocol was performed as previously described (Chapuy et al. 2013) and in accordance with the Encode guidelines (Landt et al. 2012). ChIP-seq libraries were prepared using Swift S2 Acel reagents (Swift 21096) on a Beckman Coulter Biomek i7 liquid-handling platform from ∼1 ng of DNA according to the manufacturer's protocol and using 14 cycles of PCR amplification. Sequencing libraries were quantified by Qubit fluorometer and Agilent TapeStation 2200. Library pooling and indexing were evaluated by shallow sequencing on an Illumina MiSeq. Subsequently, libraries were sequenced on an Illumina NextSeq 500 or NovaSeq 6000 by the Molecular Biology Core facilities at the Dana-Farber Cancer Institute.

For quantitative ChIP-seq analysis of H3K27 acetylation and TF binding, we used *Drosophila* chromatin/antibody spike-in control as previously described (Egan et al. 2016). For H3K27ac ChIP-seq, 4 µg of anti-H3K27ac antibody, 2 µg of spike-in antibody, and 20 ng of spike-in chromatin (Active Motif 61686 and 53083, respectively) were added to chromatin prepared from 2.5 × 10^6^ MV411 cells 72 h after RNP-mediated TF knockout. For TF ChIP-seq, 10 µg of anti-TF antibody, 5 µg of spike-in antibody, and 50 ng of spike-in chromatin were added to chromatin prepared from 100 × 10^6^ MV411 cells. The rest of the ChIP-seq experiment was performed in the standard fashion. After ChIP-seq, reads were mapped to the *Drosophila* genome and the hg38 human genome in parallel, and human tag counts were normalized to *Drosophila* tag counts.

#### HiChIP

HiChIP with primary antibodies against H3K27ac (Abcam ab4729) was performed as described (Mumbach et al. 2016; Weintraub et al. 2017). MV411 cells (three replicates of 30 × 10^6^ cells each) were cross-linked with 1% formaldehyde in PBS for 10 min at room temperature, quenched with 125 mM glycine for 5 min, washed with PBS three times, and flash-frozen. Cross-linked cell pellets were thawed on ice, resuspended in 10 µL of ice-cold Hi-C lysis buffer (10 mM Tris-HCl at pH 8.0, 10 mM NaCl, 0.2% Igepal CA-630, 1× cOmplete protease inhibitor cocktail [Roche 11697498001]), and incubated for 30 min at 4°C while rotating. Nuclei were pelleted by centrifugation at 2500*g* for 5 min at 4°C, the supernatant was discarded, and pellets were washed with 500 µL of ice-cold Hi-C lysis buffer. Nuclei were resuspended in 100 µL of 0.5% SDS, incubated for 10 min at 62°C, quenched by addition of 335 µL of 1.5% Triton X-100, and incubated for 15 min at 37°C. To digest chromatin, 50 µL of NEB buffer 2 and 375 U of MboI (NEB R0147M) were added and allowed to incubate for 2 h at 37°C while rotating, before heat inactivating for 20 min at 62°C. Sticky ends were filled in by adding 37.5 µL of 0.4 mM biotin dATP (Invitrogen 19524016), 1.5 µL of 10 mM dCTP, 1.5 µL of 10 mM dGTP, 1.5 µL of 10 mM dTTP, and 10 µL of 5 U/µL DNA polymerase I, large (Klenow) fragment (NEB M0210L), and incubating for 1 h at 37°C while rotating. Proximity ligation was performed by adding 150 µL of 10× NEB T4 ligase buffer, 125 µL of 10% Triton X-100, 7.5 µL of 20 mg/mL BSA, 10 µL of 400 U/µL T4 DNA ligase (NEB M0202L), and 655.5 µL of water and incubating for 4 h at room temperature while rotating. Proximity-ligated chromatin was sheared by sonication using a Covaris S220 sonicator in 1 mL of Covaris milliTube using the following settings: fill level 10, duty cycle 5, peak intensity power 140, cycles per burst 200, and time 6 min. Sonicated chromatin was clarified by centrifugation at 16,100*g* for 15 min at 4°C, the pellet was discarded, and the chromatin was precleared by incubation with 60 µL of Dynabeads Protein G (Invitrogen 10004D) for 1 h at 4°C while rotating. Chromatin immunoprecipitation was performed by incubating the precleared chromatin with 75 µL of Dynabeads Protein G bound with 7.5 µg of H3K27ac antibody (Abcam ab4729) overnight at 4°C while rotating. The beads were then washed twice with 1 mL of sonication buffer (50 mM HEPES-KOH at pH 7.5, 140 mM NaCl, 1 mM EDTA at pH 8.0, 1 mM EGTA at pH 8.0, 1% Triton X-100, 0.1% sodium deoxycholate, 0.1% SDS), once with 1 mL of high-salt sonication buffer (50 mM HEPES-KOH at pH 7.5, 500 mM NaCl, 1 mM EDTA at pH 8.0, 1 mM EGTA at pH 8.0, 1% Triton X-100, 0.1% sodium deoxycholate, 0.1% SDS), once with 1 mL of LiCl wash buffer (20 mM Tris-HCl at pH 8.0, 1 mM EDTA at pH 8.0, 250 mM LiCl, 0.5% Igepal CA-630, 0.5% sodium deoxycholate, 0.1% SDS), and once with 1 mL of 50 mM NaCl in TE buffer. Immunoprecipitated chromatin was eluted by resuspending the beads in ChIP elution buffer (50 mM Tris-HCl at pH 8.0, 10 mM EDTA at pH 8.0, 1% SDS) and incubating for 15 min at 65°C. Eluted chromatin was treated with 2.5 µL of 33 mg/mL RNase A (Sigma R4642) for 2 h at 37°C followed by 10 µL of 20 mg/mL proteinase K (Invitrogen 25530049) for 45 min at 55°C, and then incubated for 5 h at 65°C to reverse cross-links. DNA was purified using Zymo ChIP DNA Clean & Concentrator kit (Zymo D5205) and eluting with 14 µL of water. Five microliters of Dynabead MyOne Streptavidin C1 beads (Invitrogen 65001) was washed with 1 mL of Tween wash buffer (5 mM Tris-HCl at pH 7.5, 0.5 mM EDTA at pH 8.0, 1M NaCl, 0.05% Tween-20) and resuspended in 10 µL of 2× Biotin binding buffer (10 mM Tris-HCl at pH 7.5, 1 mM EDTA at pH 8.0, 2M NaCl). To capture biotinylated DNA, 10 µL of purified DNA was added to the beads and incubated for 15 min at room temperature with intermittent agitation, and the supernatant was discarded. Beads were washed twice with 500 µL of Tween wash buffer with shaking at 1000 rpm for 2 min at 55°C. For tagmentation, beads were resuspended in 25 µL of 2× Nextera Tagment DNA buffer (Illumina FC-121-1030), 3.25 µL of TDE1 per 50 ng of DNA, and resuspension buffer (RSB) up to 50-µL final volume, and allowed to incubate with shaking at 1000 rpm for 2 min at 55°C. The reaction was quenched by adding 500 µL of 50 mM EDTA and incubating for 30 min at 50°C. Beads were then washed twice with 500 µL of 50 mM EDTA and incubated for 3 min at 50°C, twice with 500 µL of Tween wash buffer and incubated for 2 min at 50°C, and once with 500 µL of 10 mM Tris-HCl (pH 7.5). To prepare HiChIP libraries, beads were resuspended in 15 µL of Nextera PCR Master mix, 5 µL of PCR primer cocktail, 5 µL of index primer 1, 5 µL of index primer 2, and 20 µL of water. HiChIP libraries were amplified by the following PCR program:5 min at 72°C;1 min at 98°C; 15 sec at 98°C, 30 sec at 63°C, and 1 min at 72°C, repeated for 10 cycles; 1 min at 72°C ; and held at 4°C. HiChIP libraries were purified using the Zymo DNA Clean & Concentrator kit (Zymo D4013) and eluted using 2 vol of 10 µL of water each time. Purified HiChIP libraries were subjected to 50-bp paired-end sequencing on an Illumina HiSeq 2500.

#### Micro-C

Micro-C libraries were prepared using the described protocol with minor modifications (Hsieh et al. 2020). Briefly, 5 × 10^6^ exponentially growing MV411 cells were fixed in 1% formaldehyde for 10 min at room temperature. The cross-linking reaction was quenched by adding Tris-HCl (pH 7.5) to the final concentration of 0.75 M at room temperature. Fixed cells were washed twice with 1× PBS and then subjected to the second cross-linking reaction by 3 mM DSG (Thermo Fisher 20593) for 45 min at room temperature. The DSG solution was freshly made at a 300 mM concentration in DMSO and diluted to 3 mM in PBS before use. The cross-linking reaction was quenched by 0.75 M Tris-HCl and washed twice with PBS. Cross-linked cells were snap-frozen in liquid nitrogen and stored at −80°C. Intact nuclei were extracted by treating cells with Micro-C buffer #1 (50 mM NaCl, 10 mM Tris-HCl at pH 7.5, 5 mM MgCl_2_, 1 M CaCl_2_, 0.2% NP-40, 1× protease inhibitor cocktail [Sigma 11836170001]) for 20 min on ice. Chromatin was digested with MNase (Worthington Biochem LS004798). MNase titration was performed to yield 90% monomer/10% dimers, and 10 U of MNase per 1 million cells was ultimately used. MNase digestion was performed for 10 min at 37°C and stopped by adding 4 mM EGTA for 10 min at 65°C. Digested chromatin was washed twice with ice-cold Micro-C buffer #2 (50 mM NaCl, 10 mM Tris-HCl at pH 7.5, 10 mM MgCl_2_). MNase-digested fragments were then subjected to end repairing and labeling. First, chromatin was incubated with 50 U of T4 polynucleotide kinase (NEB M0201) in Micro-C end repair buffer (50 mM NaCl, 10 mM Tris-HCl at pH 7.5, 10 mM MgCl_2_, 100 µg/mL BSA, 2 mM ATP, 5 mM DTT) for 15 min at 37°C. Second, chromatin was treated with 50 U of DNA polymerase I, large (Klenow) fragment (NEB M0210) in the absence of dNTPs for 15 min at 37°C. Third, the blunting and labeling reaction was triggered upon adding biotin-dATP (Jena Bioscience NU-835-BIO14-S), biotin-dCTP (Jena Bioscience NU-809-BIOX-S), dGTP, and dTTP to a final concentration of 66 mM each) in 1× T4 DNA ligase reaction buffer (NEB B0202). The reaction was incubated for 45 min at 25°C with interval mixing and then inactivated with 30 mM EDTA for 20 min at 65°C. Biotin-labeled chromatin was washed once by ice-cold Micro-C buffer #3 (50 mM Tris-HCl at pH 7.5, 10 mM MgCl_2_) and then subjected to the proximity ligation reaction with 10,000 U of T4 DNA ligase (NEB M0202) in 1× T4 DNA ligase reaction buffer (NEB B0202) at room temperature for at least 2.5 h with slow rotation. After ligation, biotin-dNTPs from the unligated ends were removed with 1000 U of exonuclease III (NEB M0206) in 1× NEBuffer 1 (NEB B7001) for 15 min at 37°C. Samples were then reverse cross-linked with 2 mg/mL proteinase K (Thermo Fisher 25530049), 1% SDS, and 0.1 mg/mL RNase A (Thermo Fisher EN0531) overnight at 65°C. DNA was extracted by phenol:chloroform:isoamyl alcohol (25:24:1) and ethanol precipitation. To specifically extract the ligated dinucleosomal DNA, a band at the size of 250–400 bp corresponding to the ligated dimers was gel-extracted for library preparation. The purified DNA with biotin-dNTPs was captured by Dynabeads MyOne Streptavidin C1 (Thermo Fisher Scientific 65001). Standard Illumina library preparation protocol including end repair, A-tailing, and adaptor ligation was performed on beads with the NEBnext Ultra II kit (New England Biolabs E7645). An optimal PCR cycle for final library amplification using NEBnext Ultra II Q5 was determined, and between six and nine PCR cycles were used in our study. Libraries were quantified by a Qubit fluorometer and Qubit dsDNA HS kit (Thermo Fisher Q32851). Size distribution of the libraries (∼300–500 bp) was verified by a TapeStation D1000 ScreenTape (Agilent 5067-5582). Illumina NovaSeq 50-bp paired-end sequencing (PE50) was used to obtain ∼200 million reads for each replicate.

#### RNA-seq

For RNA-seq experiments, the total cellular RNA was extracted using the QuickRNA kit (Zymo Research R1054). Purified total RNA was mixed with the ERCC ExFold RNA spike-in mix (Invitrogen 4456740). RNA sequencing libraries were prepared on a Beckman Coulter Biomek i7 liquid-handling platform using Roche Kapa mRNA HyperPrep strand-specific sample preparation kits (Roche 08098123702) from 200 ng of purified total RNA according to the manufacturer's protocol. Library quantification and Illumina sequencing were performed as described in the ChIP-seq section above.

#### Western blotting

Whole-cell lysates were prepared in RIPA buffer (Boston Bio-Products BP-115-500) with protease inhibitor cocktail (Thermo Fisher 23225). Lysates were boiled in Laemmli buffer (Bio-Rad 1610737), separated by SDS-PAGE, and transferred and blocked using standard methodology. HRP-conjugated antimouse and antirabbit IgG secondary antibodies were used for imaging (Bio-Rad 1706515 and 1706515) with an enhanced chemiluminescence substrate (PerkinElmer NEL104001EA) according to the manufacturers’ instructions.

#### Targeted TF degradation

MV411 cells were modified by CRISPR–HDR to express N-terminal FKBP12^F36V^ fusions of IRF8 and MEF2D, respectively. For each knock-in, donor DNA constructs were chemically synthesized and cloned into the pUC19 or pAAV-MCS2 plasmids obtained from Addgene. The donors included 400- to 800-bp homology arms flanking the inserted DNA sequence encoding the FKBP12^F36V^ degradation tag as well as mScarlet, 3xFlag, and HA tags. MV411 cells were electroporated with Cas9/sgRNA complexes targeting the HDR insertion site (with sgRNA protospacer sequence spanning the insertion site). Electroporation was performed using Lonza SF cell line 4D nucleofector (V4XC-2032). RNP complexes were formed by mixing 8.5 µg of TrueCut Cas9 protein v2 (Invitrogen A36499) and 120 pmol of sgRNA. Cells (0.3 × 10^6^) were washed with PBS and resuspended in 20 µL of SF cell line solution (Lonza). The cells were combined with the RNP mix and 0.6 µg of pDNA donor and electroporated using program DJ-100. After a 5- to 7-d incubation period, the cells were sorted for mScarlet fluorescence. Single clones were then obtained by single-cell dilution microwell plating and screened for bi-allelic donor insertion by PCR. Clones were validated by Western blotting and Sanger sequencing. TF degradation was induced by adding 500 nM dTAG^v^-1 as previously described (Nabet et al. 2020) followed by FACS measurement of mScarlet fluorescence and Western blotting.

#### SLAM-seq

Thiol (SH)-linked alkylation for the metabolic sequencing of RNA (SLAM-seq) was performed as described (Herzog et al. 2017). Briefly, a total of 2.5 × 10^6^ MV411 cells per replicate was incubated with 500 nM dTAG^V^-1 for 2 or 24 h. S^4^U labeling was performed by adding S^4^U to a final concentration of 100 µM for an additional hour. Cells were flash-frozen, and total RNA was extracted using Quick-RNA MiniPrep (Zymo Research) according to the manufacturer's instructions except including 0.1 mM DTT to all buffers. ERCC ExFold RNA spike-in mix (Invitrogen) was added to purified RNA. Thiol modification was performed by 10 mM iodoacetamide treatment followed by quenching with 20 mM DTT. RNA was purified by ethanol precipitation, and mRNA-seq was performed as described above.

#### Immunofluorescence with RNA FISH

Glass coverslips were coated with 0.01% poly-L-lysine solution (Sigma-Aldrich P4707) for 30 min at 37°C. Cells were plated on the precoated coverslips and grown for 24 h followed by fixation using 3.7% paraformaldehyde (VWR BT140770) in PBS for 10 min. Cells were washed with PBS twice followed by permeabilization using 70% ethanol overnight. Cells were washed with wash buffer A (20% Stellaris RNA FISH wash buffer A [Biosearch Technologies, Inc., SMF-WA1-60], 10% deionized formamide [EMD Millipore S4117]) in RNase-free water (Life Technologies AM9932) for 3 min. Coverslips were incubated in hybridization buffer (90% Stellaris RNA FISH hybridization buffer [Biosearch Technologies SMF-HB1-10], 10% deionized formamide) containing primary antibodies and 25 mM RNA probe (human MYC_intron with Quasar 570 dye; Stellaris ISMF-2066-5) for 4.5 h at 37°C in a humidified chamber. Cells were then incubated in antirabbit 488 antibody (Invitrogen A-11008) at a concentration of 1:100 in buffer A for 30 min at 37°C and then in buffer A containing the same secondary antibody plus 20 μg/mL Hoechst 33258 (Life Technologies H3569) for an additional 30 min at 37°C, followed by a 5-min wash in wash buffer B (Biosearch Technologies SMF-WB1-20). The coverslips were then mounted onto glass slides with VectaShield (VWR 101098-042) and sealed with nail polish (Electron Microscopy Science Nm 72180). Immunofluorescence without RNA FISH was performed using the same protocol except a mouse primary antibody against MEF2C was added instead of the RNA FISH probe, and an antimouse secondary antibody was used in addition to the antirabbit secondary antibody.

Images were acquired at the Harvard Center for Biological Imaging (HCBI) using lattice-based structured illumination microscopy (lattice SIM) on an Elyra 7 superresolution microscope (Carl Zeiss Microscopy) using a 63×/1.4 objective and imaged on pco.edge 4.2 sCMOS cameras (a dual-camera setup with motorized precision alignment). Image acquisition, postprocessing, and primary analysis were conducted with Carl Zeiss Microscopy ZEN software. Images were postprocessed using standard SIM settings with automatic channel alignment and subsequent maximum intensity projection.

#### Exogenous MYC expression

For exogenous MYC expression, a synthetic DNA sequence encoding MYC fused to TagBFP via a P2A linker was cloned into the pLVX-TetOne-Puro vector (Takara Bio 631849), and the construct was packaged into a lentiviral vector. MV411 cells were transduced with the lentivirus and selected with 1 µg/mL puromycin. RNP-mediated knockouts were carried out as described above. MYC expression was induced by adding 1 µg/mL doxycycline immediately after RNP electroporation and verified by Western blot and TagBFP fluorescence. Cell viability was followed by the CellTiter-Glo luminescent cell viability assay (Promega G7570).

#### Direct inhibition of enhancer activity

For direct inhibition of enhancer activity, we used the dCas9-KRAB-MeCP2 system (Yeo et al. 2018). sgRNAs targeting enhancers in the vicinity of the *IRF8* gene were cloned in a lentiviral expression vector (LRG2.1_Puro) and packaged into lentivirus. MV411 cells were first transduced with a lentiviral vector expressing dCas9-KRAB-MeCP2 and selected by incubation with 10 µg/mL blasticidin. The dCas9-KRAB-MeCP2-expressing cells were transduced with lentiviral gRNA-expressing vectors and additionally selected by incubation with 2 µg/mL puromycin for 48 h. Expression of the target gene (*IRF8*) was measured by ΔΔCt TaqMan qPCR and normalized to β-actin. Cell viability was followed by the CellTiter-Glo luminescent cell viability assay (Promega G7570).

### External data sets

RNA-seq BAM files for the BeatAML project (Tyner et al. 2018) were provided by Oregon Health and Science University and processed through the CCLE RNA processing pipeline (STAR/RSEM, described at https://github.com/broadinstitute/ccle_processing). Reads were normalized to transcripts per million (TPM) and filtered for protein-coding genes. The expression values were transformed to log_2_(TPM + 1).

H3K27ac ChIP-seq data from primary AML samples (McKeown et al. 2017) were downloaded from Sequence Read Archive (SRA) under accession number SRP103200 and processed using the AQUAS pipeline (https://github.com/kundajelab/chipseq_pipeline) with minor modifications and according to the Encode3 guidelines. We used data from 49 samples that passed our quality criteria.

Genetic dependency data are available for download at the Broad DepMap portal database (https://depmap.org/portal/download). Data release 20q1 was used for this study.

### Quantification and statistical analysis

#### ChIP-seq data analysis

Quality control, mapping, and analysis of the ChIP-seq data were performed using the nf-core pipeline (https://github.com/nf-core/chipseq). Differential binding of the same protein under two conditions was computed using the diffpeak function of the MACS2 pipeline (https://github.com/macs3-project/MACS). Spike-in-controlled experiments were mapped to the *Drosophila* genome and the hg38 human genome in parallel, and human tag counts were normalized to *Drosophila* tag counts as described.

#### HiChIP data analysis

HiChIP data sets were processed using HiC-Pro (https://github.com/nservant/HiC-Pro) with default settings. Briefly, HiChIP paired-end reads were aligned using Bowtie2 with the following parameters*:* ‐‐very-sensitive - L 30 ‐‐score-min L, -0.6, -0.2 ‐‐end-to-end ‐‐reorder. The restriction sites were obtained by scanning for MboI restriction enzyme fragments across the human genome. Valid interactions pairs (validPairs) were converted to a .HIC file using the hicpro2juicebox.sh script from the utility tool of HiC-Pro. The generated .HIC file contained interaction matrices at fragment and base pair resolutions. Interaction maps were visualized with Juicebox (https://aidenlab.org/juicebox).

#### Micro-C data analysis

Raw Micro-C sequencing data (in FastQ format) were mapped to the hg38 reference genome using BWA (https://github.com/lh3/bwa, version 0.7.17). Aligned reads were further processed using pairtools (https://github.com/mirnylab/pairtools, version 0.3.0) to obtain valid read pairs. Low-quality reads (phred score <30) and duplicate read pairs were removed using the “dedup” command from pairtools. Read pairs mapped to hg38 blacklisted regions (https://github.com/Boyle-Lab/Blacklist) were further removed. Resulting valid read pairs were used to generate .HIC files for visualization in Juicebox (https://aidenlab.org/juicebox).

#### SE calling and gene assignment

H3K27ac ChIP-seq reads were aligned to hg19 genome using BWA-ALN. Duplicate reads were removed using Picard MarkDuplicates (https://broadinstitute.github.io/picard) and Samtools (http://www.htslib.org). Fragment length was estimated using the spp package (https://cran.r-project.org/web/packages/spp/index.html). Broad peaks were called using MACS2 with a *P*-value cutoff of 1 × 10^−5^ using the estimated fragment length. For SE calling, each sample was run through ROSE2 (https://github.com/linlabbcm/rose2) excluding 2500 bp around TSSs (-t 2500) and the hg19 Encode blacklisted regions. SE regions were then merged and ROSE2 was rerun on all sample using the merged regions, producing the signal matrix, which was then normalized by median signal. Two or more replicate ChIP-seq experiments were performed for the vast majority of samples, and SE scores were averaged between the replicates. SE coordinates were then lifted to hg38 using the USCE Genome Browser LiftOver tool (http://genome.ucsc.edu/cgi-bin/hgLiftOver), and all subsequent analyses were conducted using the hg38 genome assembly.

To assign SEs to genes, we used a modified activity by contact (ABC) procedure (Fulco et al. 2019). First, we identified H3K27ac HiChIP loops with one end within 5000 bp of the transcription start site. Separately, we aggregated H3K27ac ChIP-seq and ATAC-seq activity within 2500 bp on either side of the non-TSS end of the HiC loop. We then calculated the ABC strength score as a product of the HiChIP loop frequency and the geometric mean of H3K27ac ChIP-seq and ATAC-seq activities. This procedure yielded ∼2.71 million loops, each associated with a particular gene by having one end near a TSS. We then trimmed the ABC loop set to remove (1) loops with both ends within 5000 bp of the TSS (accounting for ∼60,000 loops), (2) loops that overlapped with blacklist areas (as identified in https://github.com/Boyle-Lab/Blacklist/tree/master/lists/hg38-blacklist.v2.bed.gz, accounting for ∼10,000 loops), and (3) loops with ABC scores below the 89th percentile genome-wide (∼ 2,350,000 additional loops). Adjacent non-TSS regions were then stitched together. This resulted in 238,220 ABC regions. Subsequently, H3K27ac ChIP-seq peaks for all samples were remapped to the 238,220 ABC-defined regions using bamliquidator (https://github.com/BradnerLab/pipeline/wiki/bamliquidator). For each region, we extracted H3K27ac area under the curve in each sample and normalized by total H3K27ac across all the regions in that sample to yield a normalized enhancer signal. We then calculated the Pearson correlation coefficient between (1) normalized enhancer signals and (2) mRNA expression (in log_2_[TPM + 1]) for each ABC region, separately across the PDX and cell line sample sets. Effective ABC region/gene associations were chosen when either correlation coefficient was >0.3, resulting in a total of 23,170 associations. The ABC associations were merged with the standard proximity associations computed by ROSE2. Overall, our algorithm yielded 6868 distinct SEs assigned to 11,866 genes with an average of 3.7 gene associations per SE.

#### Identification of selective AML dependencies

Identification of selective AML dependencies was performed essentially as described (Dharia et al. 2021). Data from genome-scale CRISPR–Cas9 loss-of-function screens of 74,378 guide RNA species targeting 18,333 human genes in 769 cell lines (including 20 AML cell lines) were downloaded from the Broad DepMap portal database (https://depmap.org/portal/download). Data release 20q1 was used for this study (https://figshare.com/articles/dataset/DepMap_20Q1_Public/11791698). Gene dependency scores were calculated as previously described (Meyers et al. 2017). Briefly, the abundance of each guide RNA at the time of infection to its abundance after 21 d of cell culture was compared and aggregated into a single score per gene. The relative dropout ratio of each gene was then normalized to negative controls (score = 0, representing nonessential genes) and positive controls (score = −1, reflecting the median score of common essential genes). For each gene, the dependency probability was estimated as the likelihood that the gene represented a phenotype similar to positive controls (Dempster et al. 2019). The common essential genes were identified as those genes that were ranked in 90% of cell lines above a cutoff determined from the central minimum in the histogram of gene ranks in their 90th percentile least dependent line as previously described (Dempster et al. 2019).

To identify genetic dependencies that had skewed distributions across the cell lines screened, normLRT scores were calculated (McDonald et al. 2017). The log likelihood ratio of fitting to a skewed distribution for the dependency scores of each gene with the skew-t parametric family of skew elliptically contoured distribution for the error term. The log likelihood ratio of fitting to a normal distribution was calculated for the dependency scores of each gene. The normLRT score was twice the difference of the log of the likelihood ratio of fitting to a skewed distribution and the log of the likelihood ratio of fitting to a normal distribution. Skewed gene dependencies were defined as those with normLRT scores ≥100 and left-sided skew, as indicated by a mean gene effect score less than the median gene effect score.

To identify enriched dependencies in AML, a two-class comparison was performed between the gene effect scores for AML cell lines (in group, *n* = 20) and the remainder of all other cell lines in the screen (out group, *n* = 749) as previously described (Dharia et al. 2021). Briefly, a linear model was fitted to the gene effect scores divided in the in group and out group. Next, *t*-statistics and log odds ratios of differential gene effect were computed. Effect size was calculated as the difference in the mean gene effect dependency score in the in group compared with that in the out group. In addition to two-sided *P*-values, one-sided “left” *P*-values were calculated to identify gene dependency effects that were more negative (more dependent) in the in group than in the out group, and one-sided “right” *P*-values were calculated to identify those that were less dependent in the in group than in the out group. All *P*-values were corrected for multiple hypothesis testing using the Benjamini–Hochberg correction, and these adjusted *P*-values were reported as *q*-values. AML-enriched genetic dependencies were identified as those with a *q*-value <0.05 with a negative effect size (the mean of dependency gene effect score was more negative in the in group than in the out group).

We then used this data set to define a list of 225 selective AML dependencies by identifying genes that met all of the following criteria: (1) probability of dependency >0.5 in three or more AML cell lines, (2) not classified as a common essential gene in the screen, and (3) classified as either an enriched dependency or a skewed dependency.

#### RNA-seq data analysis

RNA-seq data were processed using the CCLE workflow of STAR (v2.6.1c) + RSEM (v1.3.0) with hg38 reference genome, enriched with ERCC92 v29 reference (https:// storage.googleapis.com/ccle_default_params/Homo_sapiens_assembly38_ERCC92.fasta; https://storage.googleapis.com/ccle_default_ params/STAR_genome_GRCh38_noALT_noHLA_noDecoy_ERCC_ v29_oh100.tar.gz; https://storage.googleapis.com/ccle_default_params/rsem_reference_GRCh38_gencode29_ercc.tar.gz) and Gencode v29 reference gene regions (https://storage.googleapis.com/ccle_default_params/references_gtex_gencode.v29.GRCh38.ERCC.genes.collapsed_only.gtf). Scaling factors were computed from ERCC spike-ins using the ERCCdashboard R package (https://bioconductor.org/packages/release/bioc/html/erccdashboard.html). Scaling was applied only to samples where ERCC displayed a mean scaling of at least twice the size of standard error. Differential analysis of RNA-seq data was performed using DESeq2 (v1.26.0; https://bioconductor.org/packages/release/bioc/html/DESeq2.html) using the ERCC pseudogenes to rescale the data by using the run_estimate_size_factors control_genes parameter.

#### SLAM-seq data analysis

A modified version of the slamdunk pipeline was used for SLAM-seq processing (available at https://github.com/jkobject/slamdunk). Differential analysis of SLAM-seq data was performed using DESeq2 on the TC converted transcripts (tccounts) and total read counts (totalcounts). First, mean totalcounts were used to compute scaling factors via the DESeq2 run_estimate_size_factors geoMean*s* parameter. Then, the tccounts and totalcounts were normalized to the ERCC pseudogene counts using DEseq2 getSizeFactors and setSizeFactors functions. For further details and code, see https://github.com/jkobject/AMLproject/blob/master/notebooks/slamseq_iBet_spikeIn_maxp_paper.ipynb.

#### SIM microscopy

The ZEN machine learning package was used for image processing (https://www.zeiss.com/microscopy/int/products/microscope-software/zen.html#downloads). We used ZEN 3.0 Black for image processing, including channel alignment, SIM processing, and image subsetting, and ZEN 3.1 Blue for data segmentation and formatting. The segmentation (puncta recognition) algorithm was trained on a subset of raw images labeled manually.

First, 2D (discoid) puncta of individual *z*-stacks were aggregated into 3D spheroids if the distance between the centers of the discoid puncta was less than the size of an average discoid. Puncta present only in one *z*-stack were discarded. In the rare cases when more than three RNA FISH foci were detected per nucleus due to background noise, only the top three in terms of total fluorescence were counted, as MV411 cells carry three copies of the MYC gene.

The colocalization enrichment was computed by Fisher's exact test between the expected number of red (RNA FISH) puncta colocalizing with the green (protein IF) versus the actual measured number. The expected (random) distribution of puncta was computed by defining the total volume of the nucleus occupied by the green puncta.

The image processing pipeline is available at https://github.com/jkobject/AMLproject/tree/master/notebooks/Fish_Super Res.ipynb.

#### Myeloid differentiation index

To identify markers of myeloid development, genome-wide mRNA expression values in “HSC” and “monocyte” samples from Corces et al. (2016) were processed to yield mean and variance of expression by gene. For each gene, the two variances were pooled [pooled variance = mean (HSC variance, monocyte variance)]. A separation index was then defined for each gene as the difference between the HSC and monocyte mean expressions divided by the square root of the pooled variance. Markers were chosen as the 19 genes with the highest separation indices: MS4A6A, ACPP, TGFBI, IL1RN, CHST15, CTSS, CD300C, CD1D, KIT, ABLIM1, CD34, NLRC3, ALDH5A1, RBPMS, ATP8B2, PROM1, MEIS1, CMBL, and BAALC. Lymphoid markers were identified with the same procedure, comparing HSC samples with the T-cell and B-cell samples. To compute the myeloid index, each sample's expression of the marker genes as determined above was converted to a *z*-score using the mean of all eight HSC and monocyte expressions and the pooled variance for that gene, and further normalized to a ±1 scale by dividing by the maximum absolute value of all *z*-scores. For genes where the mean monocyte score was higher than the mean HSC score, normalized scores were multiplied by −1. The normalized scores for the set of marker genes were summed to define the myeloid index for each sample. The same procedure was used for the lymphoid index, with appropriate cell type substitution. Plotting normal and leukemia cell types according to the myeloid and lymphoid indices yielded the expected development vectors as shown in Supplemental Figure S13.

### Data and software availability

Raw sequencing data have been deposited to the SRA database under accession number PRJNA751732. ChIP-seq data from pediatric AML samples are in the Gene Expression Omnibus database under accession number GSE155558. A complete list of deposited raw and processed data, as well as QC, is in Supplemental Data 1. Links to code are in the relevant sections of the Materials and Methods.

Key resources are in Supplemental Data 1. Cell line, PDX, and primary sample characteristics are in Supplemental Data 2. The SE matrix is in Supplemental Data 3. AML dependencies are in Supplemental Data 4. Changes in mRNA expression after TF knockouts are in Supplemental Data 5. SLAM-seq after MEF2D degradation is in Supplemental Data 6. SLAM-seq after IRF8 degradation is in Supplemental Data 7.

## Supplementary Material

Supplemental Material
