# Supplementary material for: A distinct core regulatory module enforces oncogene expression in KMT2A-rearranged leukemia
Source: Genes Dev. 2022 Mar 1;36(5-6):368–89. doi: 10.1101/gad.349284.121 (PMC8973843; doi:10.1101/gad.349284.121)
Supplement: Supplemental Material [file supp_36_5-6_368__DC1.html]

Supplemental Material 

# A distinct core regulatory module enforces oncogene expression in KMT2A-rearranged leukemia

## Supplemental Material

- Supplemental\_Figures\_and\_Legends.pdf
- Supplemental\_Data.zip
