# Supplementary material for: A distinct core regulatory module enforces oncogene expression in KMT2A-rearranged leukemia
Source: Genes Dev. 2022 Mar 1;36(5-6):368–89. doi: 10.1101/gad.349284.121 (PMC8973843; doi:10.1101/gad.349284.121)
Supplement: Supplemental Material [file supp_gad.349284.121_Supplemental_figures_and_Legends.pdf]

Figure S1

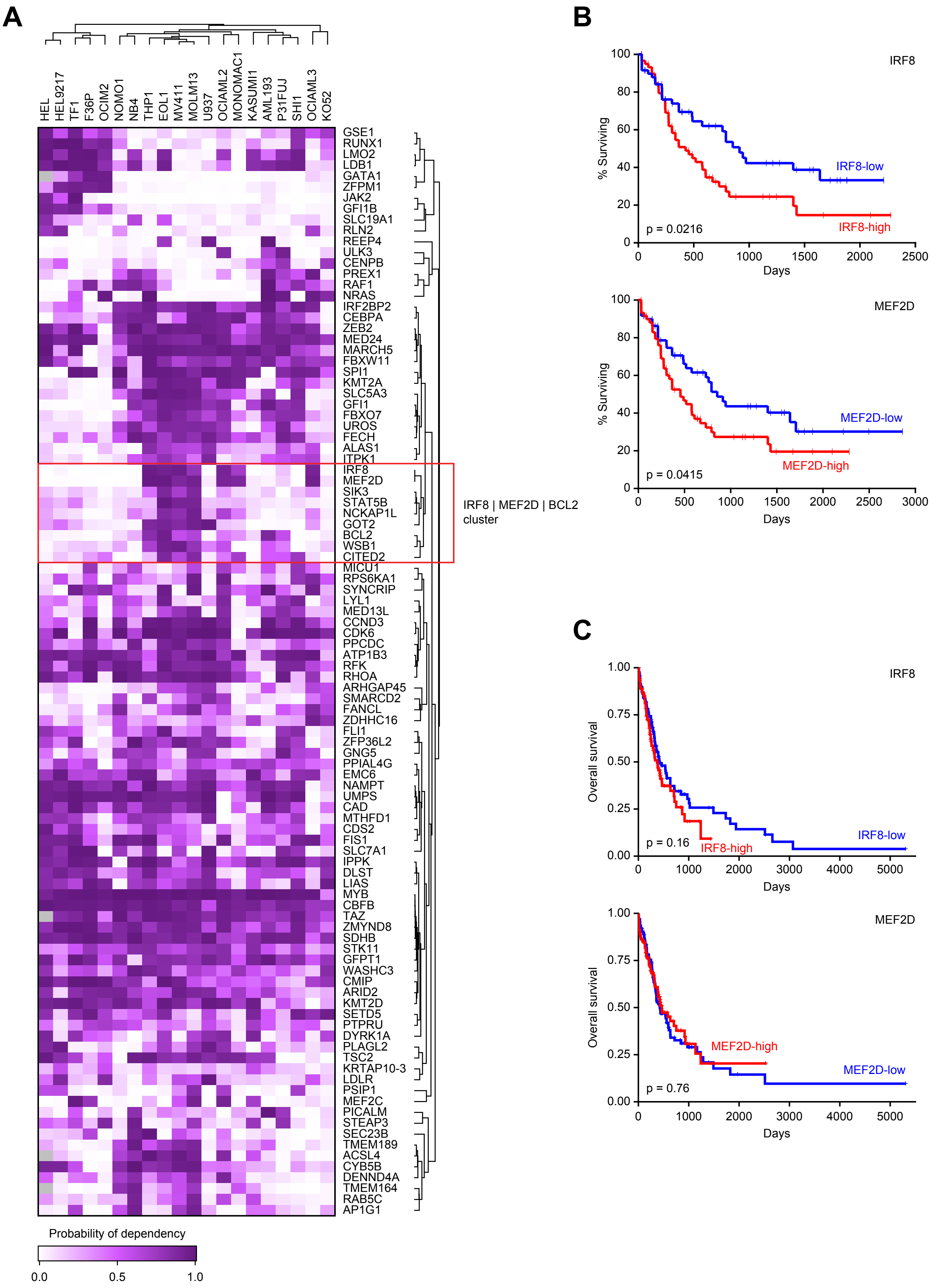

A
HEL
HEL9217
TF1
F36P
OCIM2
NOMO1
NB4
THP1
EOL1
MV411
MOLM13
U937
OCIAML2
MONOMAC1
KASUMI1
AML193
P31FUJ
SHI1
OCIAML3
KO52
GSE1
RUNX1
LMO2
LDB1
GATA1
ZFPM1
JAK2
GFI1B
SLC19A1
RLN2
REEP4
ULK3
CENPB
PREX1
RAF1
NRAS
IRF2BP2
CEBPA
ZEB2
MED24
MARCH5
FBXW11
SPI1
KMT2A
SLC5A3
GFI1
FBXO7
UROS
FECH
ALAS1
ITPK1
IRF8
MEF2D
SIK3
STAT5B
NCKAP1L
GOT2
BCL2
WSB1
CITED2
MICU1
RPS6KA1
SYNCRIP
LYL1
MED13L
CCND3
CDK6
PPCDC
ATP1B3
RFK
RHOA
ARHGAP45
SMARCD2
FANCL
ZDHHC16
FLI1
ZFP36L2
GNG5
PPIAL4G
EMC6
NAMPT
UMPS
CAD
MTHFD1
CDS2
FIS1
SLC7A1
IPPK
DLST
LIAS
MYB
CBFB
TAZ
ZMYND8
SDHB
STK11
GFPT1
WASHC3
CMIP
ARID2
KMT2D
SETD5
PTPRU
DYRK1A
PLAGL2
TSC2
KRTAP10-3
LDLR
PSIP1
MEF2C
PICALM
STEAP3
SEC23B
TMEM189
ACSL4
CYB5B
DENND4A
TMEM164
RAB5C
AP1G1
IRF8 | MEF2D | BCL2
cluster
Probability of dependency
0.0
0.5
1.0
B
IRF8
% Surviving
100
80
60
40
20
0
IRF8-low
IRF8-high
p = 0.0216
0
500
1000
1500
2000
2500
Days
MEF2D
% Surviving
MEF2D-low
MEF2D-high
p = 0.0415
0
500
1000
1500
2000
2500
3000
Days
C
IRF8
Overall survival
1.00
0.75
0.50
0.25
0.00
p = 0.16
IRF8-high
IRF8-low
0
1000
2000
3000
4000
5000
Days
MEF2D
Overall survival
MEF2D-high
MEF2D-low
p = 0.76
Days

Figure S1, related to Figure 1. Selective AML gene dependencies

A, A heatmap of 225 selective AML dependencies reflecting probability of dependency inferred from the skewed LRT scores (Methods). Rows and columns are hierarchically clustered on Pearson correlation with complete linkage.

B, Kaplan-Meyer plots of overall survival in TCGA AML patients (Network 2013) with MEF2D and IRF8 expression above the 60th percentile (red line) and below the 40th percentile (blue line).

C, Kaplan-Meyer plots of overall survival in BeatAML patients (Tyner et al. 2018) with MEF2D and IRF8 expression above the 60th percentile (red line) and below the 40th percentile (blue line).

Figure S2

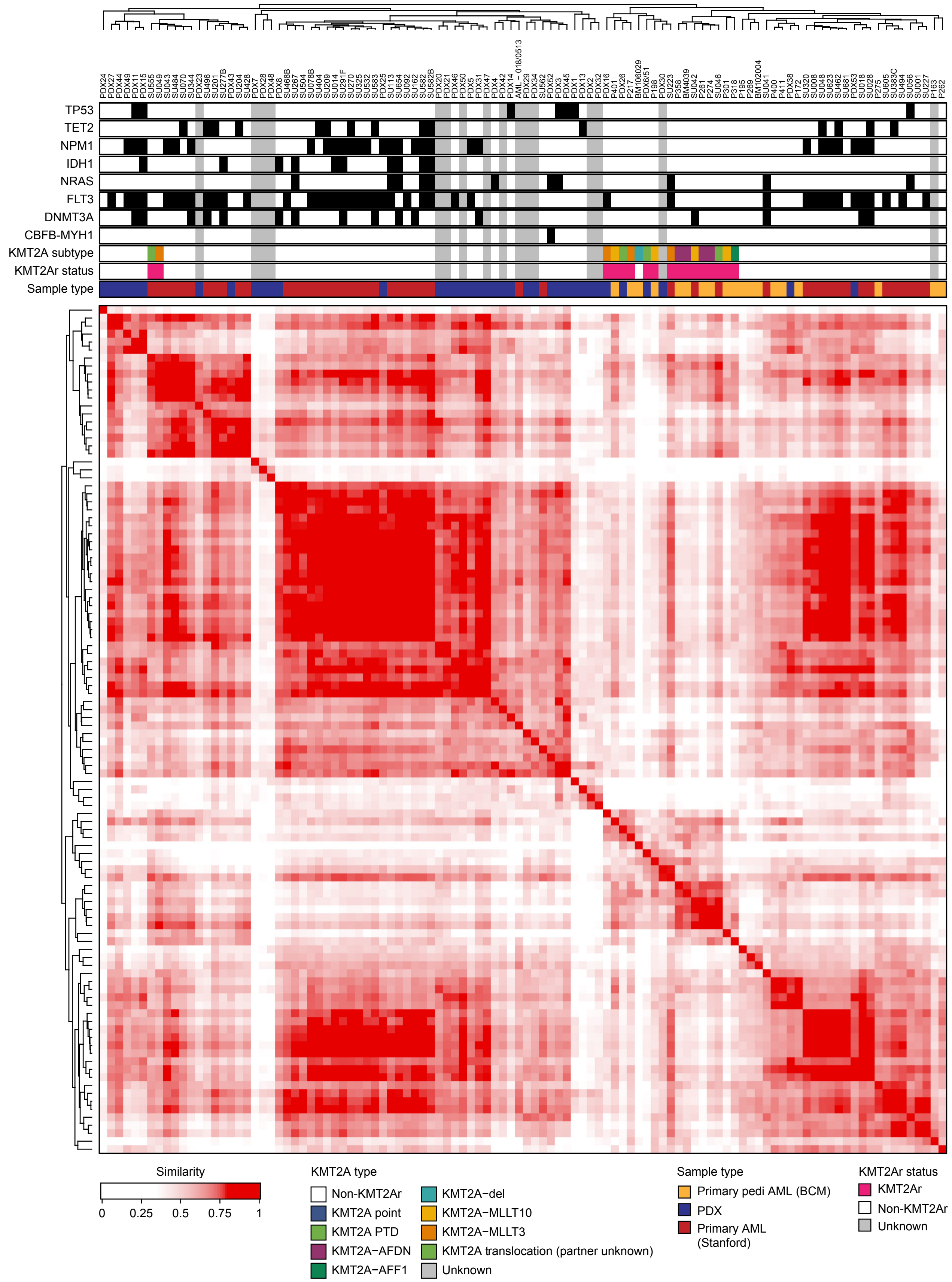

TP53
TET2
NPM1
IDH1
NRAS
FLT3
DNMT3A
CBFB-MYH1
KMT2A subtype
KMT2Ar status
Sample type
Similarity
0
0.25
0.5
0.75
1
KMT2A type
Non-KMT2Ar
KMT2A point
KMT2A PTD
KMT2A−AFDN
KMT2A−AFF1
KMT2A−del
KMT2A−MLLT10
KMT2A−MLLT3
KMT2A translocation (partner unknown)
Unknown
Sample type
Primary pedi AML (BCM)
PDX
Primary AML (Stanford)
KMT2Ar status
KMT2Ar
Non-KMT2Ar
Unknown

Figure S2, related to Figure 2. A superenhancer-based classification of AML: primary samples and PDX models. The heatmap is a similarity matrix based on AML classification in Figure 2B. Primary and PDX samples are hierarchically clustered with complete linkage using Pearson correlation of the scores of 4798 superenhancers recurrent in at least 2 samples. The bars at the top reflect presence of coding mutations (grey color designates unknown) and sample type.

Figure S3

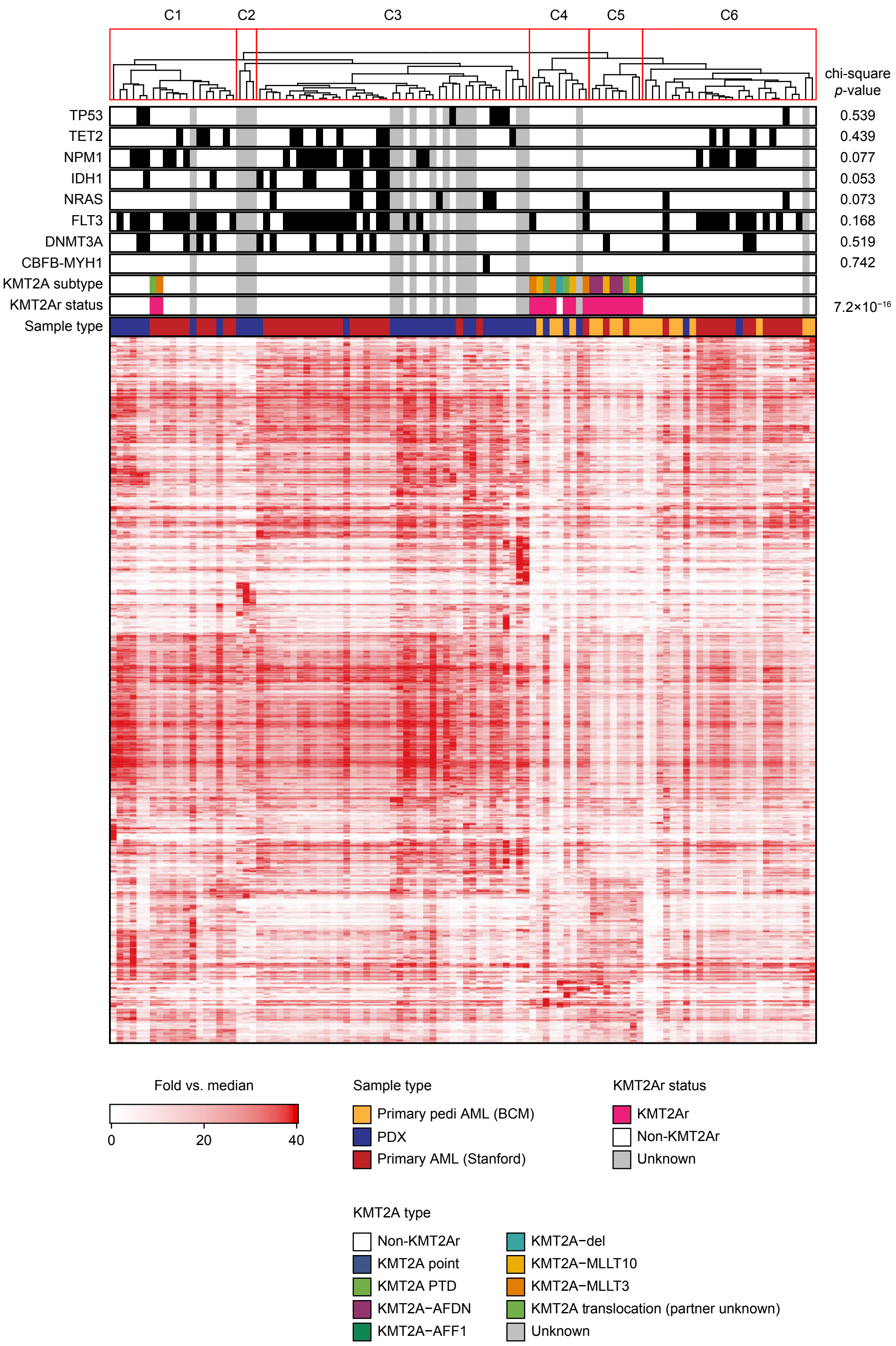

C1
C2
C3
C4
C5
C6
chi-square
p-value
TP53
TET2
NPM1
IDH1
NRAS
FLT3
DNMT3A
CBFB-MYH1
KMT2A subtype
KMT2Ar status
Sample type
0.539
0.439
0.077
0.053
0.073
0.168
0.519
0.742
7.2×10⁻¹⁶
Fold vs. median
0
20
40
Sample type
Primary pedi AML (BCM)
PDX
Primary AML (Stanford)
KMT2Ar status
KMT2Ar
Non-KMT2Ar
Unknown
KMT2A type
Non-KMT2Ar
KMT2A point
KMT2A PTD
KMT2A−AFDN
KMT2A−AFF1
KMT2A−del
KMT2A−MLLT10
KMT2A−MLLT3
KMT2A translocation (partner unknown)
Unknown

Figure S3, related to Figure 2. A superenhancer-based classification of AML: primary samples and PDX models. The heatmap is a signal matrix (a full version of the AML classification in Figure 2B). Primary and PDX samples are hierarchically clustered with complete linkage using Pearson correlation of the scores of 4798 superenhancers recurrent in at least 2 samples, forming 6 distinct clusters. The bars at the top reflect presence of coding mutations (grey color designates unknown) and sample type. The side plot reflects *p*-values of non-random distribution of somatic mutations across the 6 clusters, calculated by the one-sided chi-square test, demonstrating that KMT2A rearrangements are the only mutation that shows significant enrichment in any cluster ($p<0.05$).

Figure S4

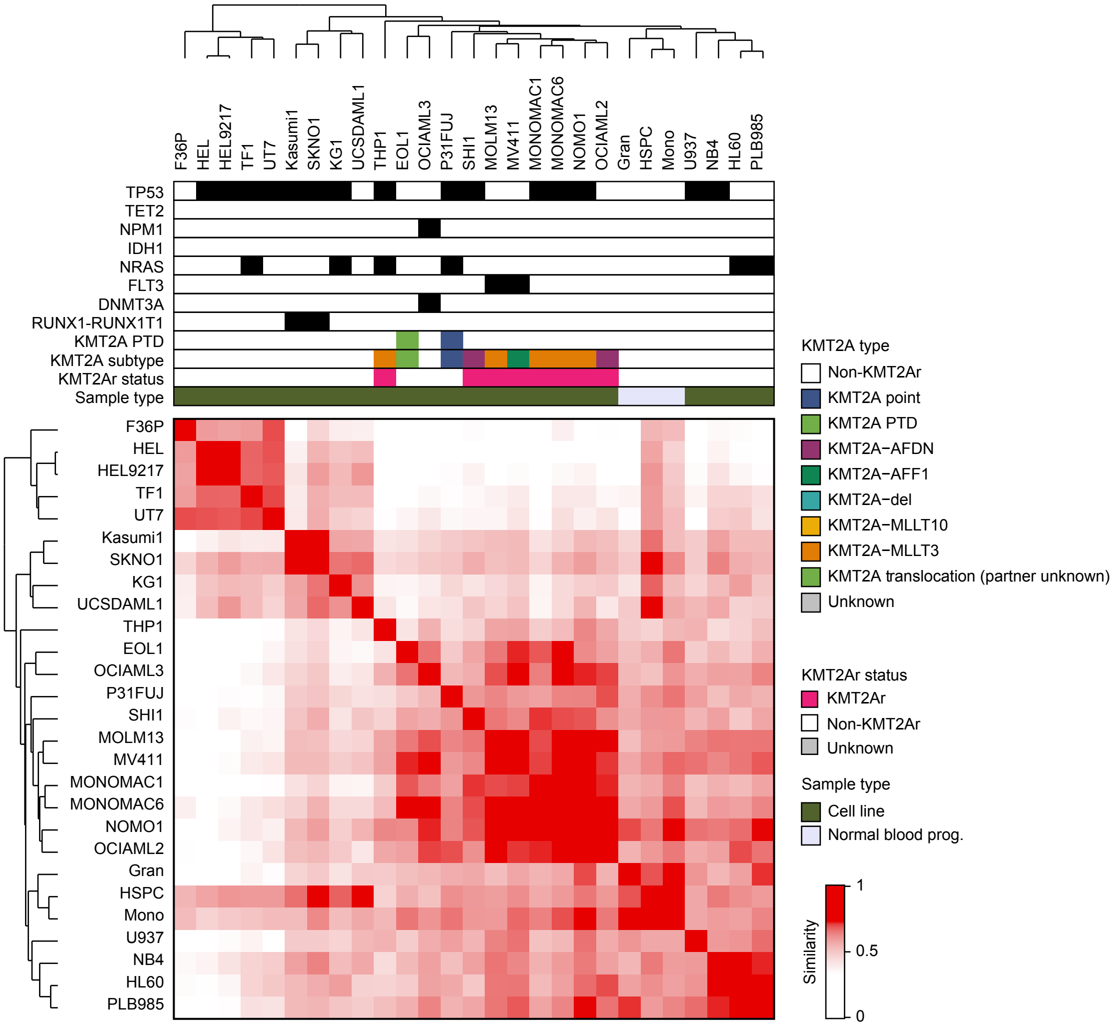
F36P
HEL
HEL9217
TF1
UT7
Kasumi1
SKNO1
KG1
UCSDAML1
THP1
EOL1
OCIAML3
P31FUJ
SHI1
MOLM13
MV411
MONOMAC1
MONOMAC6
NOMO1
OCIAML2
Gran
HSPC
Mono
U937
NB4
HL60
PLB985
TP53
TET2
NPM1
IDH1
NRAS
FLT3
DNMT3A
RUNX1-RUNX1T1
KMT2A PTD
KMT2A subtype
KMT2Ar status
Sample type
KMT2A type
Non-KMT2Ar
KMT2A point
KMT2A PTD
KMT2A−AFDN
KMT2A−AFF1
KMT2A−del
KMT2A−MLLT10
KMT2A−MLLT3
KMT2A translocation (partner unknown)
Unknown
KMT2Ar status
KMT2Ar
Non-KMT2Ar
Unknown
Sample type
Cell line
Normal blood prog.
Similarity
1
0.5
0

Figure S4, related to Figure 2. A superenhancer-based classification of AML: cell lines. The heatmap is a similarity matrix of the cell lines clustered with complete linkage using Pearson correlation of the scores of 4798 superenhancers recurrent in at least 2 samples. The heatmap reflects pairwise Pearson correlation of superenhancer scores. The bars at the top reflect presence of coding mutations and sample type.

Figure S5

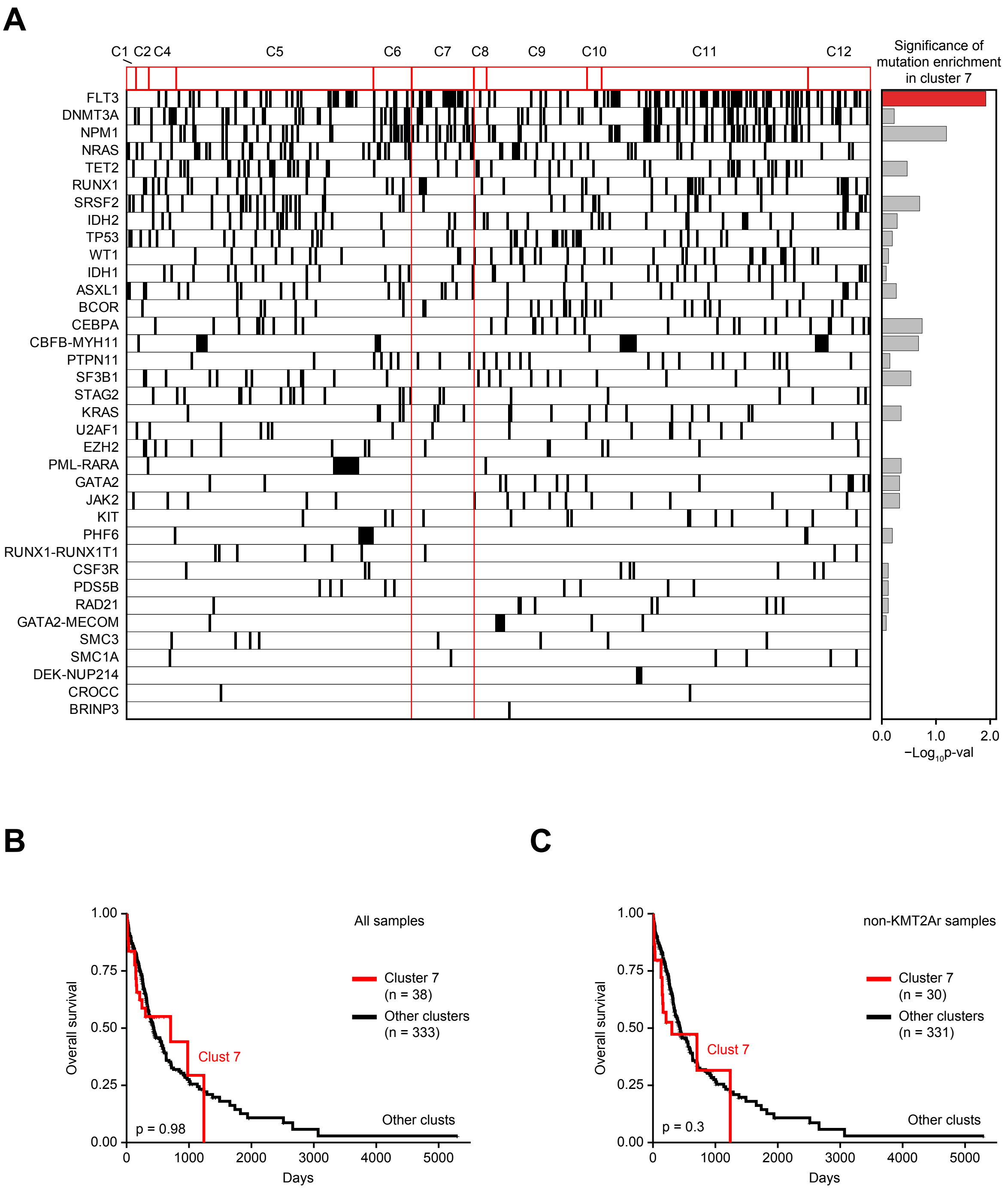

A
C1 C2 C4 C5 C6 C7 C8 C9 C10 C11 C12
Significance of mutation enrichment in cluster 7
FLT3
DNMT3A
NPM1
NRAS
TET2
RUNX1
SRSF2
IDH2
TP53
WT1
IDH1
ASXL1
BCOR
CEBPA
CBFB-MYH11
PTPN11
SF3B1
STAG2
KRAS
U2AF1
EZH2
PML-RARA
GATA2
JAK2
KIT
PHF6
RUNX1-RUNX1T1
CSF3R
PDS5B
RAD21
GATA2-MECOM
SMC3
SMC1A
DEK-NUP214
CROCC
BRINP3
0.0 1.0 2.0
−Log10p-val
B
All samples
Cluster 7 (n = 38)
Other clusters (n = 333)
Clust 7
p = 0.98
Other clusts
Overall survival
Days
C
non-KMT2Ar samples
Cluster 7 (n = 30)
Other clusters (n = 331)
Clust 7
p = 0.3
Other clusts
Overall survival
Days

Figure S5, related to Figures 2 and 3. Mutational landscape and survival profile of KMT2Ar-like AML.

A, Enrichment of somatic mutations in KMT2Ar-like AMLs from the BeatAML cluster 7 (defined by mRNA expression of core regulatory TFs; Figure 3A). The bar plot demonstrates *p*-values calculated by chi-square test of mutation distribution between KMT2Ar-negative samples in cluster 7 (*i.e.* KMT2Ar-like) versus KMT2Ar-negative samples in all other clusters. Only *FLT3* mutations are significantly over-represented in cluster 7, with an enrichment value of 1.7-fold compared to randomly expected distribution. The 38 most common point mutations and translocations from the BeatAML study were included in this analysis and are visualized in the order of decreasing overall frequency. Patients with KMT2Ar AML were excluded from the analysis.

B, Kaplan-Meyer plot of overall survival of the BeatAML patients (Tyner et al. 2018), demonstrating no significant difference in survival of patients from cluster 7, including KMT2Ar and KMT2Ar-like AML, compared to other clusters.

C, Kaplan-Meyer plot of overall survival in BeatAML patients (Tyner et al. 2018), demonstrating no significant difference in survival of KMT2Ar-like AML patients from cluster 7 compared to other clusters. KMT2Ar AMLs were excluded from the analysis.

Figure S6

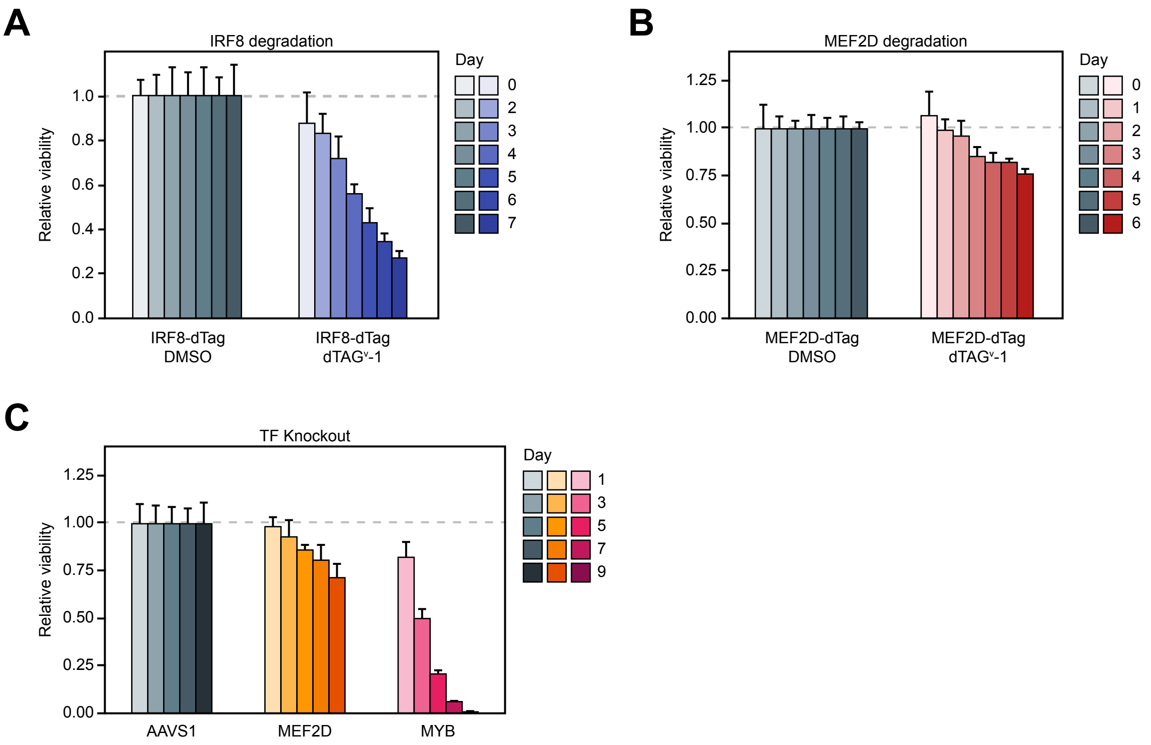

A
IRF8 degradation
Relative viability
0.0
0.2
0.4
0.6
0.8
1.0
IRF8-dTag
DMSO
IRF8-dTag
dTAGᵛ-1
Day
0
2
3
4
5
6
7
B
MEF2D degradation
Relative viability
0.00
0.25
0.50
0.75
1.00
1.25
MEF2D-dTag
DMSO
MEF2D-dTag
dTAGᵛ-1
Day
0
1
2
3
4
5
6
C
TF Knockout
Relative viability
0.00
0.25
0.50
0.75
1.00
1.25
AAVS1
MEF2D
MYB
Day
1
3
5
7
9

Figure S6, related to Figures 5-7. Application of targeted TF degradation to elucidate direct transcriptional effects of MEF2D and IRF8.

A, Targeted degradation of IRF8 results in loss of cell viability. Cells carrying a fusion-based IRF8 degron were incubated with DMSO *vs.* dTAG^{V}-1 and cell viability was followed by quantification of ATP pools using a luciferin-based assay.

B, Targeted degradation of MEF2D results in loss of cell viability. The experiment was carried out as in (B).

C, CRISPR/Cas9 knockout of MEF2D results in a loss of cell viability that is quantitatively similar to the effects of MEF2D degradation. MYB knockout was used as positive control.

Figure S7

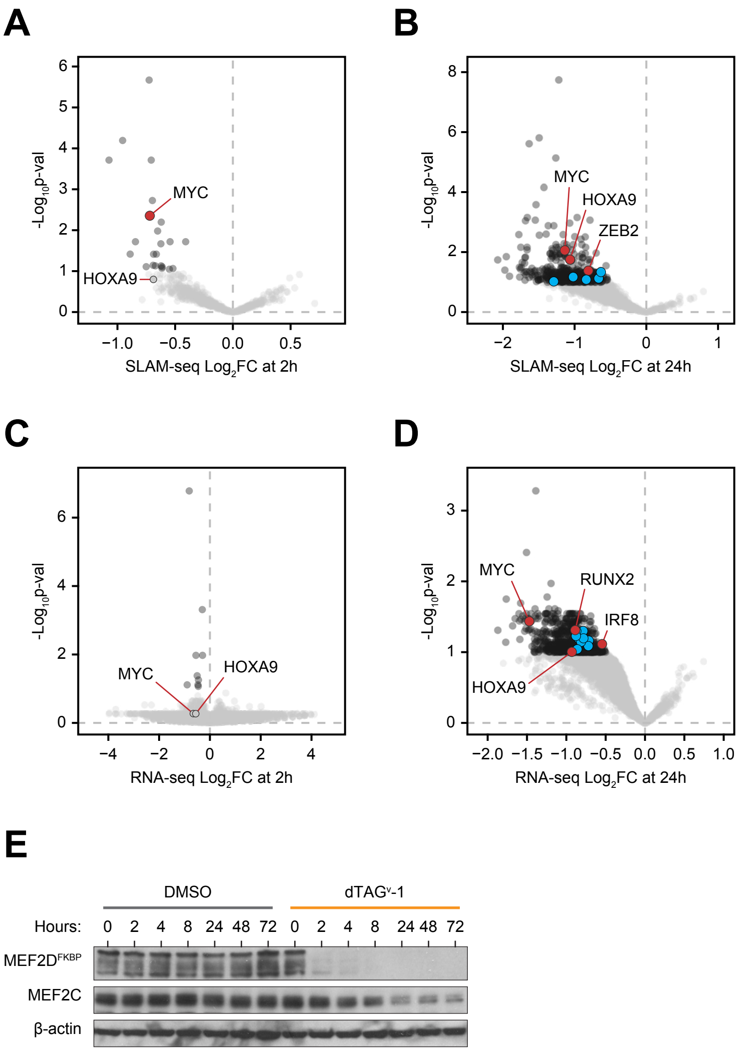
A
B
C
D
E
MYC
HOXA9
ZEB2
RUNX2
IRF8
-Log10p-val
SLAM-seq Log2FC at 2h
SLAM-seq Log2FC at 24h
RNA-seq Log2FC at 2h
RNA-seq Log2FC at 24h
DMSO
dTAGV-1
Hours:
0 2 4 8 24 48 72 0 2 4 8 24 48 72
MEF2DFKBP
MEF2C
β-actin

Figure S7, related to Figure 5. Volcano plots of SLAM-seq and mRNA-seq following MEF2D degradation, and regulation of MEF2C by MEF2D. Core regulatory TFs are highlighted in blue.

A, SLAM-seq 2 hours after MEF2D degradation.

B, SLAM-seq 24 hours after MEF2D degradation.

C, mRNA-seq 2 hours after MEF2D degradation.

D, mRNA-seq 24 hours after MEF2D degradation.

E, Western blot showing reduced MEF2C levels after prolonged MEF2D degradation.

Figure S8

A

						2 hrs SLAM-seq			24 hrs SLAM-seq			2 hrs RNA-seq			24 hrs RNA-seq		
Gene ID	MV411 dep. probability	MV411 drop out score	Pan-lethal	SE in MV411	TF	log_2FC	pvalue	padj	log_2FC	pvalue	padj	log_2FC	pval	padj	log_2FC	pval	padj
FUT4	0.009	-9E-04	FALSE	1		-0.725	2.6E-09	2.1E-06	-0.933	0.002	0.043	-0.452	2.9E-04	0.232	-0.757	0.004	0.089
ZFP36	0.008	0.012	FALSE	1		-0.955	1.6E-07	6.4E-05	-1.630	3.2E-09	2.4E-06	-0.659	0.038	0.534	-1.477	0.025	0.124
BHLHE40	0.001	0.141	FALSE	1	1	-0.707	8.3E-07	1.9E-04	-1.035	0.006	0.062	-0.500	0.018	0.534	-1.019	0.002	0.072
PTGER4	0.001	0.143	FALSE	1		-1.073	9.5E-07	1.9E-04	-1.128	0.009	0.069	-0.944	0.003	0.446	-1.086	0.003	0.082
KLF10	0.002	0.101	FALSE		1	-0.697	1.2E-05	0.002	-0.697	0.048	0.143	-0.545	0.009	0.521	-0.757	0.073	0.167
MYC	1.000	-1.935	TRUE	1	1	-0.720	3.3E-05	0.004	-1.136	1.4E-04	0.009	-0.655	0.075	0.534	-1.470	2.7E-04	0.037
F3	0.141	-0.259	FALSE			-0.620	5.5E-05	0.006	-0.828	0.010	0.070	-0.541	0.064	0.534	-0.953	0.002	0.076
TNFAIP3	0.003	0.093	FALSE			-0.652	1.0E-04	0.010	-0.681	0.093	0.209	-0.520	0.006	0.503	-1.051	4.9E-04	0.046
TXNIP	0.438	-0.449	FALSE			-0.544	2.4E-04	0.019	-1.039	6.0E-04	0.019	-0.468	0.058	0.534	-0.960	0.001	0.058
PMAIP1	4E-04	0.252	FALSE	1		-0.844	2.3E-04	0.019	-0.962	0.003	0.046	-0.785	8.5E-04	0.299	-1.277	0.002	0.065
PIM1	0.001	0.138	FALSE	1		-0.409	2.6E-04	0.019	-0.813	0.003	0.047	-0.455	0.008	0.521	-0.895	0.001	0.055
IER2	0.003	0.072	FALSE	1		-0.624	3.2E-04	0.021	-1.219	8.0E-12	1.8E-08	-0.459	4.7E-05	0.086	-1.275	5.6E-04	0.047
ID2	0.204	-0.309	FALSE	1		-0.890	6.1E-04	0.038	-1.545	3.5E-06	8.4E-04	-0.905	0.024	0.534	-1.212	0.007	0.097
TP53RK	0.908	-0.843	TRUE	1		-0.663	6.6E-04	0.038	-0.880	0.017	0.090	-0.478	1.1E-04	0.164	-0.799	0.002	0.072
KLF6	5E-04	0.222	FALSE		1	-0.687	7.1E-04	0.039	-1.656	2.4E-06	6.9E-04	-0.590	0.035	0.534	-1.293	6.7E-04	0.049
KLF11	0.109	-0.228	FALSE		1	-0.691	1.4E-03	0.072	-1.325	0.003	0.046	-0.706	0.006	0.498	-0.975	8.4E-04	0.052
CDKN1B	0.006	0.035	FALSE	1		-0.671	1.6E-03	0.074	-1.308	2.1E-04	0.011	-0.751	0.062	0.534	-1.478	6.3E-04	0.049
CDC42EP3	0.012	-0.023	FALSE	1		-0.622	1.6E-03	0.074	-0.980	0.002	0.041	-0.584	0.008	0.521	-1.041	5.3E-04	0.047
ZBTB33	0.032	-0.103	FALSE	1	1	-0.751	1.8E-03	0.078	-1.280	0.009	0.069	-0.637	0.041	0.534	-0.945	0.147	0.255
RANBP6	0.038	-0.120	FALSE			-0.612	1.9E-03	0.079	-0.990	0.027	0.113	-0.477	0.011	0.534	-0.705	0.035	0.129
CPEB2	2E-04	0.293	FALSE	1		-0.615	2.2E-03	0.085	-1.259	5.8E-05	0.005	-0.890	0.002	0.376	-1.474	1.7E-04	0.033
SATB2	0.063	-0.168	FALSE	1	1	-0.519	2.3E-03	0.086	-1.119	0.007	0.062	-0.369	2.9E-04	0.232	-0.766	0.007	0.099
DUSP6	0.005	0.037	FALSE	1		-0.550	2.5E-03	0.090	-0.914	0.005	0.056	-0.695	0.055	0.534	-1.520	1.5E-04	0.033

B

						2 hrs SLAM-seq			24 hrs SLAM-seq			2 hrs RNA-seq			24 hrs RNA-seq		
Gene ID	MV411 dep. probability	MV411 drop out score	Pan-lethal	SE in MV411	TF	log_2FC	pvalue	padj	log_2FC	pvalue	padj	log_2FC	pval	padj	log_2FC	pval	padj
MYC	1.000	-1.935	TRUE	1	1	-0.720	3.3E-05	0.004	-1.136	1.4E-04	0.009	-0.655	0.075	0.534	-1.470	2.7E-04	0.037
LMO2	0.013	-0.032	FALSE	1	1	-0.446	0.006	0.138	-0.770	0.037	0.128	-0.171	0.001	0.334	-0.479	0.017	0.116
ZEB2	0.931	-0.914	FALSE	1	1	-0.425	0.008	0.152	-0.809	0.002	0.041	-0.322	0.010	0.527	-0.877	0.001	0.059
HOXA9	0.305	-0.374	FALSE	1	1	-0.677	0.011	0.172	-1.063	5.5E-04	0.018	-0.539	0.043	0.534	-0.930	0.008	0.099
MYB	0.999	-1.468	FALSE	1	1	-0.356	0.047	0.307	-1.020	0.008	0.068	-0.440	0.056	0.534	-0.782	0.001	0.064
IRF8	0.946	-0.984	FALSE	1	1	-0.279	0.091	0.376	-0.837	0.014	0.083	-0.019	0.803	0.929	-0.546	0.003	0.077
RUNX1	0.167	-0.281	FALSE	1	1	-0.195	0.152	0.480	-0.671	0.041	0.134	-0.487	0.004	0.450	-0.861	0.005	0.091
E2F3	0.200	-0.306	FALSE	1	1	-0.234	0.176	0.499	-0.498	0.098	0.213	-0.444	0.001	0.299	-0.840	0.002	0.065
MEIS1	0.376	-0.414	FALSE	1	1	-0.274	0.184	0.513	-0.865	0.037	0.128	-0.184	0.222	0.574	-0.757	0.010	0.106
ETV6	1E-04	0.378	FALSE	1	1	-0.160	0.296	0.614	-0.569	0.084	0.197	-0.029	0.767	0.916	-0.563	0.011	0.108
GFI1	0.916	-0.864	FALSE	1	1	-0.123	0.288	0.614	-0.665	0.011	0.076	-0.172	0.036	0.534	-0.714	0.002	0.069
RUNX2	0.132	-0.250	FALSE	1	1	-0.151	0.312	0.614	-0.810	0.027	0.114	-0.393	0.005	0.492	-0.887	6.4E-04	0.049
PLAGL2	0.316	-0.380	FALSE		1	-0.149	0.383	0.665	-0.444	0.094	0.209	-0.161	0.009	0.521	-0.720	0.003	0.082
SP1	0.864	-0.764	FALSE		1	-0.156	0.395	0.672	-0.653	0.038	0.130	-0.127	0.164	0.544	-0.624	0.024	0.123
MEF2C	0.523	-0.496	FALSE	1	1	-0.205	0.428	0.693	-0.835	0.037	0.130	-0.369	0.045	0.534	-0.750	0.020	0.120
CEBPA	0.485	-0.475	FALSE	1	1	0.127	0.484	0.718	-0.293	0.137	0.257	0.103	0.377	0.687	-0.389	0.073	0.167
RXRA	0.582	-0.530	FALSE	1	1	0.099	0.555	0.761	-0.371	0.248	0.366	0.104	0.249	0.597	-0.300	0.225	0.344
ZMYND8	0.920	-0.875	FALSE	1		-0.059	0.738	0.884	-0.629	0.041	0.134	-0.058	0.754	0.915	-0.786	7.6E-04	0.050
MEF2D	0.877	-0.784	FALSE	1	1	-0.018	0.913	0.962	-0.632	0.003	0.046	0.021	0.823	0.939	-0.444	0.060	0.153
MAX	0.395	-0.425	TRUE		1	0.179	0.460		-0.708	0.074	0.183	-0.026	0.738	0.906	-0.471	0.033	0.128
FLI1	0.318	-0.381	FALSE	1	1	-0.167	0.537		-1.290	0.019	0.096	-0.289	0.060	0.534	-0.571	0.047	0.139
SPI1	0.943	-0.967	FALSE	1	1	-0.182	0.603		0.038	0.938	0.951	0.236	0.050	0.534	-0.286	0.198	0.314
STAT5B	0.898	-0.820	FALSE		1	0.572	0.143		-0.287	0.661		0.110	0.261	0.604	-0.306	0.114	0.215
SREBF1	0.898	-0.820	FALSE	1	1	0.657	0.280		-0.133	0.813		0.243	0.180	0.549	-0.131	0.561	0.667
TFAP4	0.941	-0.959	FALSE		1	0.089	0.833		-0.636	0.473		-0.064	0.587	0.824	-0.624	0.018	0.116
ZNF281	0.074	-0.185	FALSE		1	-0.160	0.638		-0.746	0.099	0.214	-0.557	0.146	0.538	-1.042	0.021	0.121
LYL1	0.142	-0.260	FALSE	1	1	0.401	0.437		0.018	0.966	0.974	0.243	0.081	0.534	-0.197	0.425	0.543
FOSL2	0.400	-0.428	FALSE	1	1	0.019	0.944		-0.553	0.163	0.284	-0.358	0.012	0.534	-0.812	0.002	0.072
GATA2	2E-04	0.296	FALSE	1	1	0.112	0.667		-1.029	0.047	0.141	-0.080	0.342	0.660	-0.627	0.024	0.123
HHEX	0.051	-0.149	FALSE		1	-0.344	0.201		-1.312	0.032	0.121	-0.495	0.007	0.521	-0.871	7.8E-04	0.050
ZFPM1	0.017	-0.053	FALSE	1	1	-1.127	0.253		-0.428	0.671		0.519	0.030	0.534	-0.345	0.262	0.383

Figure S8, related to Figure 5. A table summary of SLAM-seq and RNA-seq data following MEF2D degradation

A, Genes demonstrating a statistically significant change in transcription rate (SLAM-seq adjusted *p*-value <0.1) at 2 hours following MEF2D degradation.

B, Changes in core regulatory TF transcription after MEF2D degradation.

Figure S9

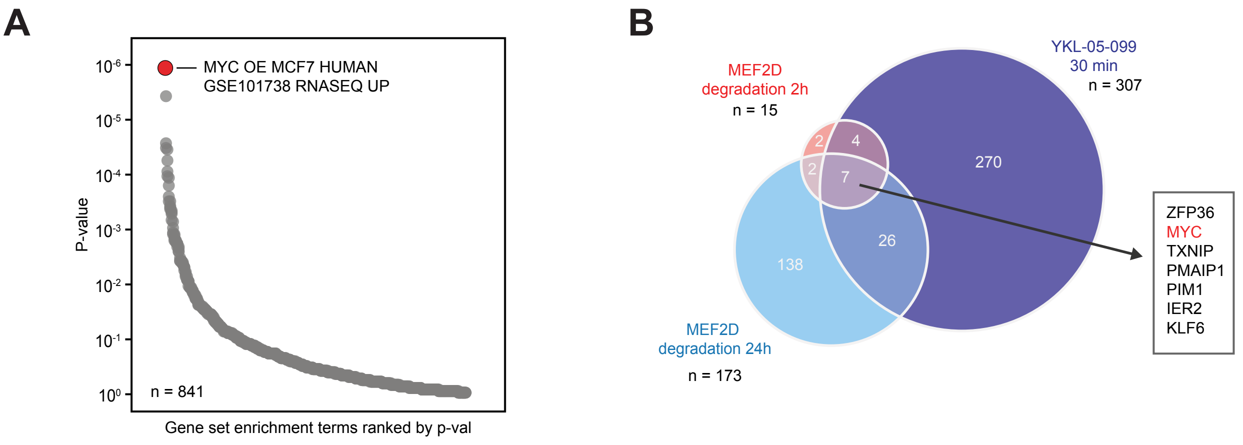

C

JQ1, µM

YKL-05-009, nM	12.5	6.25	3.125	1.563	0.781	0.391
400	0.05	0.091	0.022	-0.05	0.05	0.031
200	0.08	0.136	0.018	0.058	0.064	0.06
100	0.049	0.48	0.059	-0	0.14	0.076
50	0.029	0.024	0.043	0.015	0.064	0.06
25	0.057	0.045	0.039	-0	0.117	0.136
12.5	0.049	0.068	0.07	0.011	0.069	0.072

D

MYCi361, µM

YKL-05-009, nM	3.125	1.563	0.781	0.391	0.195	0.098
400	-0.05	0.03	0.089	0.102	0.133	0.032
200	-0.04	0.095	0.091	0.145	0.095	0.034
100	0.033	0.016	0.037	0.079	0.15	0.072
50	-0.04	-0.02	0.252	0.063	0.042	0.05
25	0.04	0.039	0.093	0.06	0.059	0.038
12.5	0.034	0.005	0.029	0.023	0.044	0.052

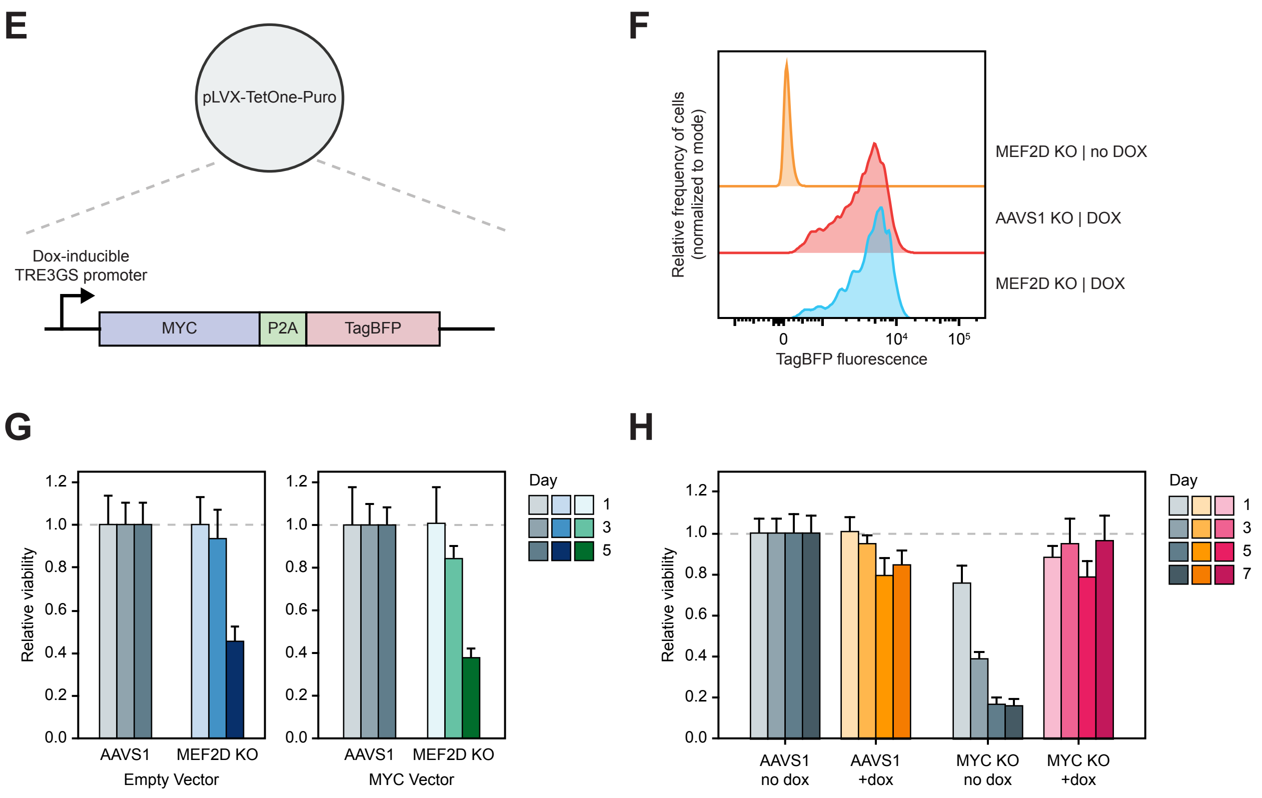

Figure S9, related to Figure 5. A functional interaction between MYC and MEF2D

A, Gene set enrichment analysis of the genes showing significant changes in transcription rates after MEF2D degradation using Ehrichr, filtered to display only human-derived data (Kuleshov et al. 2016).

B, Intersection of gene sets demonstrating significant changes in transcription (adjusted *p*-value <0.05) measured by SLAM-seq after MEF2D degradation and indirect MEF2C/D inhibition by YKL-05-099 treatment.

C, Excess over bliss synergy matrix demonstrating synergy between SIK/MEF2 inhibitor YKL-05-099 and bromodomain inhibitor JQ1. Red color demonstrates synergy.

D, Excess over bliss synergy matrix demonstrating synergy between SIK/MEF2 inhibitor YKL-05-099 and MYC inhibitor MYCi361. Red color demonstrates synergy.

E, Cloning strategy for a doxycycline-inducible lentiviral vector expressing MYC-P2A-TagBFP.

F, Induction of MYC expression demonstrated by TagBFP fluorescence after addition of doxycycline.

G, Forced exogenous expression of MYC fails to rescue the cell viability loss following MEF2D degradation.

H, As a positive control, forced exogenous expression of MYC completely rescues MYC knockout by CRISPR/Cas9. Exogenous MYC expression was induced by doxycycline.

Figure S10

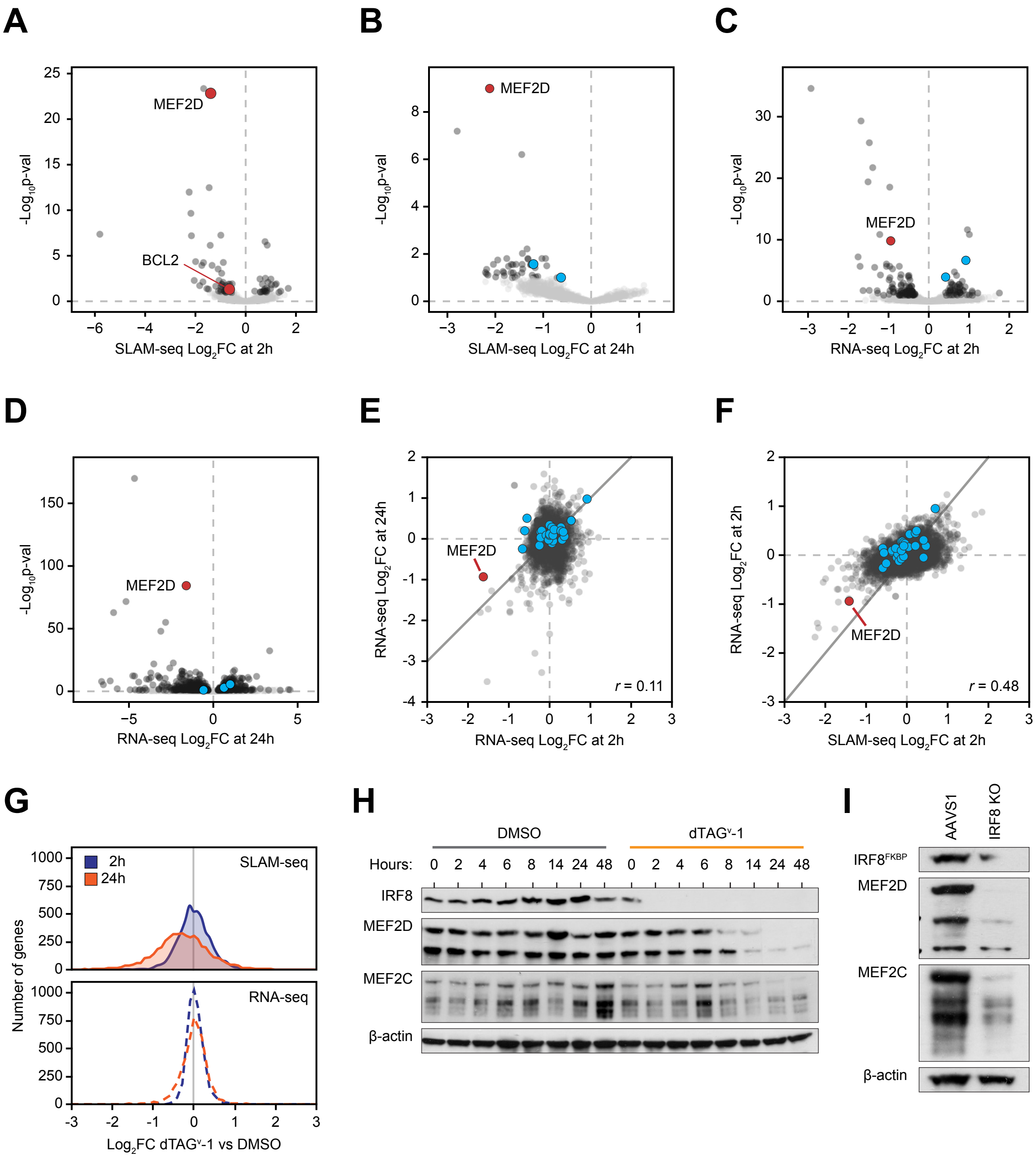
A
MEF2D
BCL2
-Log10p-val
SLAM-seq Log2FC at 2h
B
MEF2D
SLAM-seq Log2FC at 24h
C
MEF2D
RNA-seq Log2FC at 2h
D
MEF2D
RNA-seq Log2FC at 24h
E
MEF2D
RNA-seq Log2FC at 24h
RNA-seq Log2FC at 2h
r = 0.11
F
MEF2D
RNA-seq Log2FC at 2h
SLAM-seq Log2FC at 2h
r = 0.48
G
2h
24h
SLAM-seq
RNA-seq
Number of genes
Log2FC dTAGV-1 vs DMSO
H
DMSO
dTAGV-1
Hours: 0 2 4 6 8 14 24 48 0 2 4 6 8 14 24 48
IRF8
MEF2D
MEF2C
β-actin
I
AAVS1
IRF8 KO
IRF8FKBP
MEF2D
MEF2C
β-actin

Figure S10, related to Figure 7. Targeted degradation of IRF8

A, Volcano plot of SLAM-seq 2 hours after IRF8 degradation.

B, Volcano plot of SLAM-seq 24 hours after IRF8 degradation. Core regulatory TFs are highlighted in blue.

C, Volcano plot of RNA-seq 2 hours after IRF8 degradation. Core regulatory TFs are highlighted in blue.

D, Volcano plot of RNA-seq 24 hours after IRF8 degradation. Core regulatory TFs are highlighted in blue.

E, A cross-plot of genome-wide changes in mRNA pools measured after 2 *vs.* 24 hours of IRF8 degradation demonstrates a poor correlation between early and late transcriptional response.

F, A cross-plot of genome-wide changes in mRNA pools (mRNA-seq) *vs.* transcription rates (SLAM-seq) measured after 2 hours of IRF8 degradation.

G, A distribution plot of genome-wide changes in nascent transcription rates (SLAM-seq) and mRNA pools (RNA-seq) after 2 and 24 hours of IRF8 degradation.

H, Western blot showing reduced MEF2C and MEF2D levels after IRF8 degradation.

I, Western blot showing reduced MEF2C and MEF2D levels 24 hours after IRF8 knockout.

Figure S11

Gene ID	MV411 dep. probability	MV411 drop out score	2 hrs SLAM-seq log$_2$FC	2 hrs SLAM-seq pvalue	2 hrs SLAM-seq padj	24 hrs SLAM-seq log$_2$FC	24 hrs SLAM-seq pvalue	24 hrs SLAM-seq padj	2 hrs RNA-seq log$_2$FC	2 hrs RNA-seq pval	2 hrs RNA-seq padj	24 hrs RNA-seq log$_2$FC	24 hrs RNA-seq pval	24 hrs RNA-seq padj
CXorf21	0.027	-0.089	-1.672	9.9E-28	4.4E-24	-2.795	6.0E-11	6.6E-08	-0.971	1.4E-22	2.9E-19	-1.824	1.8E-04	0.009
MEF2D	0.877	-0.784	-1.393	6.7E-27	1.5E-23	-2.118	4.6E-13	1.0E-09	-0.946	1.2E-13	1.5E-10	-1.603	6.8E-89	5.0E-85
FAM107B	4E-04	0.237	-1.453	2.2E-16	3.2E-13	-1.241	0.002	0.113	-0.690	8.4E-06	0.003	-0.771	0.028	0.296
CCR2	0.268	-0.351	-2.247	8.7E-16	9.6E-13	-3.651	0.001		-1.684	8.2E-34	5.0E-30	-1.842	2.0E-13	6.3E-11
DCANP1	0.187	-0.296	-2.179	2.2E-13	1.9E-10	-2.188	0.003		-1.394	6.2E-26	1.9E-22	-1.295	2.7E-10	4.8E-08
KCNA3	0.006	0.033	-5.798	5.2E-11	3.8E-08	-5.960	1.8E-06		-2.926	2.1E-39	2.6E-35	-5.905	3.3E-67	1.2E-63
CBX6	0.007	0.017	-0.989	7.7E-11	4.9E-08	-1.325	6.2E-05	0.015	-0.378	0.002	0.145	-0.971	0.005	0.111
TIFAB	0.007	0.016	-2.156	1.0E-10	5.6E-08	-4.186	0.003		-1.478	4.2E-30	1.7E-26	-1.755	1.6E-19	1.6E-16
LDLR	0.579	-0.528	0.770	5.0E-10	2.5E-07	-0.116	0.617	0.859	0.957	1.2E-15	2.1E-12	0.132	0.351	0.800
MALAT1			0.856	1.4E-09	6.0E-07	-0.220	0.432	0.760	0.562	0.018	0.404	-0.296	0.477	0.855
CCR1	0.019	-0.061	-1.410	1.6E-09	6.3E-07	-1.636	2.3E-04	0.028	-0.902	7.6E-09	4.9E-06	-1.001	0.001	0.042
CPM	0.007	0.020	-1.991	1.0E-07	3.8E-05	-1.815	1.6E-02		-0.494	1.3E-05	3.4E-03	-1.606	7.2E-16	4.1E-13
IKZF1	0.434	-0.447	-0.889	1.4E-07	4.8E-05	-0.977	0.008	0.175	-0.532	3.9E-04	0.045	-0.301	0.261	0.735
SERPINB8	0.006	0.029	-1.678	1.6E-07	4.9E-05	-1.501	0.045		-0.693	1.2E-04	0.019	-1.033	0.026	0.286
POU2F2	8E-05	0.393	-1.745	3.6E-07	1.0E-04	-2.154	0.001	0.076	-1.510	1.6E-23	3.9E-20	-3.523	5.7E-26	9.2E-23
ABHD15	0.069	-0.178	-1.336	3.6E-07	1.0E-04	-1.321	0.043		-0.494	2.4E-07	1.1E-04	-0.628	2.5E-05	0.002
IL6R	0.021	-0.067	-1.098	9.6E-07	2.5E-04	-0.493	0.352	0.717	-0.517	1.4E-07	7.0E-05	-0.457	0.056	0.407
TMEM127	0.698	-0.605	-0.955	3.0E-06	7.4E-04	-1.121	0.029	0.314	-0.345	8.0E-04	0.077	-0.140	0.360	0.804
TRIB1	0.005	0.045	0.783	1.1E-05	0.003	-0.084	0.703	0.897	0.687	1.1E-07	5.7E-05	-0.075	0.750	0.947
SCD	0.919	-0.871	0.566	1.4E-05	0.003	-0.344	0.289	0.665	0.040	0.691	0.912	0.256	0.119	0.561
HMGCS1	0.965	-1.096	1.003	1.5E-05	0.003	-0.891	0.033	0.335	0.784	1.1E-05	0.003	-0.378	0.074	0.462
OPN3	0.002	0.128	-2.046	2.3E-05	0.004	-2.730	0.034		-0.561	1.9E-04	0.027	-0.488	0.103	0.531
ADGRG5	0.016	-0.044	-1.060	2.3E-05	0.004	-1.347	0.104		-0.579	2.6E-04	0.034	-0.288	0.008	0.148
CSF1R	0.229	-0.326	-1.702	3.5E-05	0.006	-1.519	0.109		-0.705	3.1E-07	1.3E-04	-0.881	0.009	0.158
MSMO1	0.002	0.110	0.887	4.0E-05	0.007	-1.452	0.008	0.187	1.004	9.2E-15	1.3E-11	0.107	0.278	0.748
TGFBR1	0.003	0.078	-0.899	4.2E-05	0.007	-1.727	3.1E-04	0.032	-0.332	0.079	0.649	-0.718	0.060	0.420
P2RY2	0.006	0.025	-0.538	6.6E-05	0.010	-0.101	0.723	0.904	-0.429	1.6E-04	0.024	0.068	0.643	0.916
DDIT3	0.160	-0.275	1.216	6.5E-05	0.010	-0.146	0.678	0.884	0.330	0.028	0.476	-0.153	0.452	0.845
ZBTB33	0.032	-0.103	-0.944	7.6E-05	0.011	-1.126	0.004	0.149	-0.902	3.4E-05	0.008	-1.022	0.005	0.112
B3GNT7	0.004	0.056	-0.684	7.6E-05	0.011	-0.416	0.270	0.646	-0.634	2.7E-06	9.7E-04	-0.229	0.008	0.140
CALR	0.040	-0.125	0.663	7.8E-05	0.011	0.583	0.096	0.477	0.093	0.447	0.820	0.623	2.6E-04	0.011
DUSP7	0.004	0.056	-0.633	8.4E-05	0.012	-0.518	0.326	0.694	-0.138	0.222	0.721	-0.656	0.058	0.417
INHBA	0.012	-0.023	-0.939	9.7E-05	0.013	-1.763	5.9E-05	0.015	-0.277	0.083	0.650	-1.321	2.1E-09	3.2E-07
JUN	0.374	-0.413	1.026	1.1E-04	0.013	0.314	0.272	0.646	0.765	2.4E-05	0.005	-0.152	0.584	0.895
284454			1.170	1.0E-04	0.013	0.681	0.023	0.298	0.639	0.042	0.548	0.039	0.871	0.979
SH2B3	0.002	0.107	-0.791	1.3E-04	0.016	-1.384	0.124	0.503	-0.461	3.6E-04	0.042	-0.374	0.274	0.746
LYZ	0.041	-0.126	-1.829	1.4E-04	0.017	-0.611	0.503		-0.275	0.044	0.556	0.546	0.135	0.588
TIFA	0.003	0.093	-1.191	1.6E-04	0.019	-1.692	0.012	0.210	-0.653	3.6E-04	0.042	-1.095	0.003	0.076
ZNF616	4E-04	0.249	-0.983	1.7E-04	0.019	-0.824	0.450		-0.213	0.223	0.721	0.232	0.363	0.806
RNF41	0.193	-0.301	-0.678	1.8E-04	0.020	-0.898	0.050	0.368	-0.183	0.074	0.644	-0.223	0.028	0.293
EGR1	0.007	0.017	0.837	2.9E-04	0.031	-0.124	0.578	0.845	0.606	8.9E-05	0.017	-0.342	0.240	0.715
HELQ	0.005	0.039	-0.792	3.1E-04	0.032	-0.613	0.173	0.564	-0.351	0.012	0.344	-0.180	0.562	0.887
BIN3-IT1			1.691	3.2E-04	0.033	0.252	0.652	0.872	0.828	0.025	0.453	-0.098	0.817	0.965
MBP	0.002	0.112	-0.650	3.5E-04	0.035	-0.755	0.010	0.205	-0.385	0.015	0.383	-0.184	0.377	0.813
ZNF765	3E-04	0.288	-1.544	3.6E-04	0.035	-0.895	0.492		-0.265	0.250	0.736	0.067	0.889	0.982
KLF2	0.002	0.121	0.989	3.7E-04	0.035	0.307	0.239	0.625	0.833	6.8E-05	0.014	-0.585	0.023	0.266
HES6	0.010	-0.009	0.760	3.9E-04	0.037	0.523	0.199	0.580	0.281	0.187	0.703	0.065	0.845	0.972
RHOU	0.030	-0.099	-1.481	4.3E-04	0.039	0.229	0.771		-0.475	0.003	0.188	0.131	0.627	0.911
TMED10	0.483	-0.473	-0.780	4.7E-04	0.042	-0.838	0.120	0.497	-0.254	0.093	0.653	-0.215	0.439	0.839
BCL2	0.576	-0.526	-0.649	5.4E-04	0.048	-1.205	0.005	0.159	-0.340	0.038	0.534	-0.713	3.2E-07	3.4E-05
FLVCR2	0.034	-0.110	-0.988	5.7E-04	0.048	-1.404	0.012	0.211	-0.418	1.2E-04	0.019	-0.426	9.7E-05	0.005
CRKL	0.902	-0.830	-0.611	5.6E-04	0.048	-0.039	0.967	0.986	-0.155	0.240	0.732	0.192	0.614	0.906
HNRNPA3	0.138	-0.256	0.733	6.3E-04	0.053	0.223	0.478	0.791	-0.119	0.464	0.825	-0.133	0.604	0.902
LRRC8C	0.041	-0.128	-0.817	6.8E-04	0.055	-2.041	1.8E-04	0.027	-0.366	0.060	0.611	-1.075	0.015	0.208

Figure S11 cont.

			2 hrs SLAM-seq			24 hrs SLAM-seq			2 hrs RNA-seq			24 hrs RNA-seq		
Gene ID	MV411 dep. probability	MV411 drop out score	log_2FC	pvalue	padj	log_2FC	pvalue	padj	log_2FC	pval	padj	log_2FC	pval	padj
LINC00599			0.849	7.3E-04	0.059	0.435	0.169	0.562	0.725	0.212	0.715	0.685	0.010	0.167
NLRP3	0.002	0.130	-1.080	8.1E-04	0.063	-1.668	1.8E-04	0.027	-0.688	7.2E-04	0.071	-0.959	1.4E-08	2.0E-06
ZNF189	5E-04	0.229	-0.858	8.2E-04	0.063	-1.239	0.036	0.347	-0.349	0.056	0.603	0.110	0.787	0.960
GLUL	0.002	0.122	0.357	9.0E-04	0.066	0.521	0.058	0.383	0.141	0.191	0.708	1.087	1.1E-05	8.1E-04
FADS1	0.006	0.034	0.710	9.1E-04	0.066	-0.743	0.145	0.531	-0.093	0.419	0.809	0.232	0.022	0.261
MIR9-3HG			0.780	9.1E-04	0.066	-0.337	0.355	0.720	0.125	0.467	0.826	0.078	0.607	0.903
GORASP1	0.091	-0.207	1.392	8.9E-04	0.066	0.727	0.366		0.303	0.040	0.542	0.998	2.1E-11	4.9E-09
ZNF766	0.001	0.215	-0.591	9.6E-04	0.068	-0.855	0.020	0.270	0.040	0.735	0.925	0.120	0.454	0.846
TMEM70	0.087	-0.203	-0.996	0.001	0.072	-1.093	0.029	0.312	-0.213	0.248	0.735	-0.138	0.544	0.880
GNA13	0.003	0.073	-0.838	0.001	0.072	-1.282	0.011	0.208	-0.540	0.008	0.291	-0.629	0.242	0.717
KCNQ1OT1			0.770	0.001	0.073	0.088	0.823	0.946	0.808	0.003	0.193	-0.002	0.994	0.999
RGS16	0.003	0.080	0.657	0.001	0.078	-0.996	0.005	0.149	0.694	0.001	0.100	-0.649	0.123	0.570
INSIG1	0.005	0.046	0.584	0.001	0.079	-0.431	0.227	0.609	0.705	0.000	0.004	0.100	0.727	0.940
RCSD1	0.060	-0.164	-0.778	0.001	0.080	-0.990	0.027	0.310	-0.279	0.018	0.405	-0.022	0.923	0.987
HNRNPA2B1	0.314	-0.379	0.585	0.001	0.086	0.488	0.189	0.571	-0.034	0.775	0.938	0.109	0.420	0.832
CXCL8	0.028	-0.094	0.819	0.001	0.086	-0.134	0.722	0.904	0.898	1.6E-05	0.004	0.062	0.902	0.984
DMXL2	0.091	-0.207	1.322	0.001	0.090	0.189	0.723	0.904	-0.009	0.952	0.990	0.547	0.222	0.699
BNIP2	0.060	-0.164	-0.792	0.002	0.093	-0.811	0.025	0.298	-0.523	0.015	0.388	-0.325	0.350	0.799
RAB11FIP1	3E-04	0.279	-0.560	0.002	0.097	-0.832	0.002	0.113	-0.352	4.7E-04	0.052	-0.210	0.221	0.697

Figure S11, related to Figure 7. A table summary of SLAM-seq and RNA-seq data following IRF8 degradation. Genes demonstrating a statistically significant change in transcription rate (SLAM-seq adjusted p-value <0.1) at 2 hours following IRF8 degradation are presented.

Figure S12

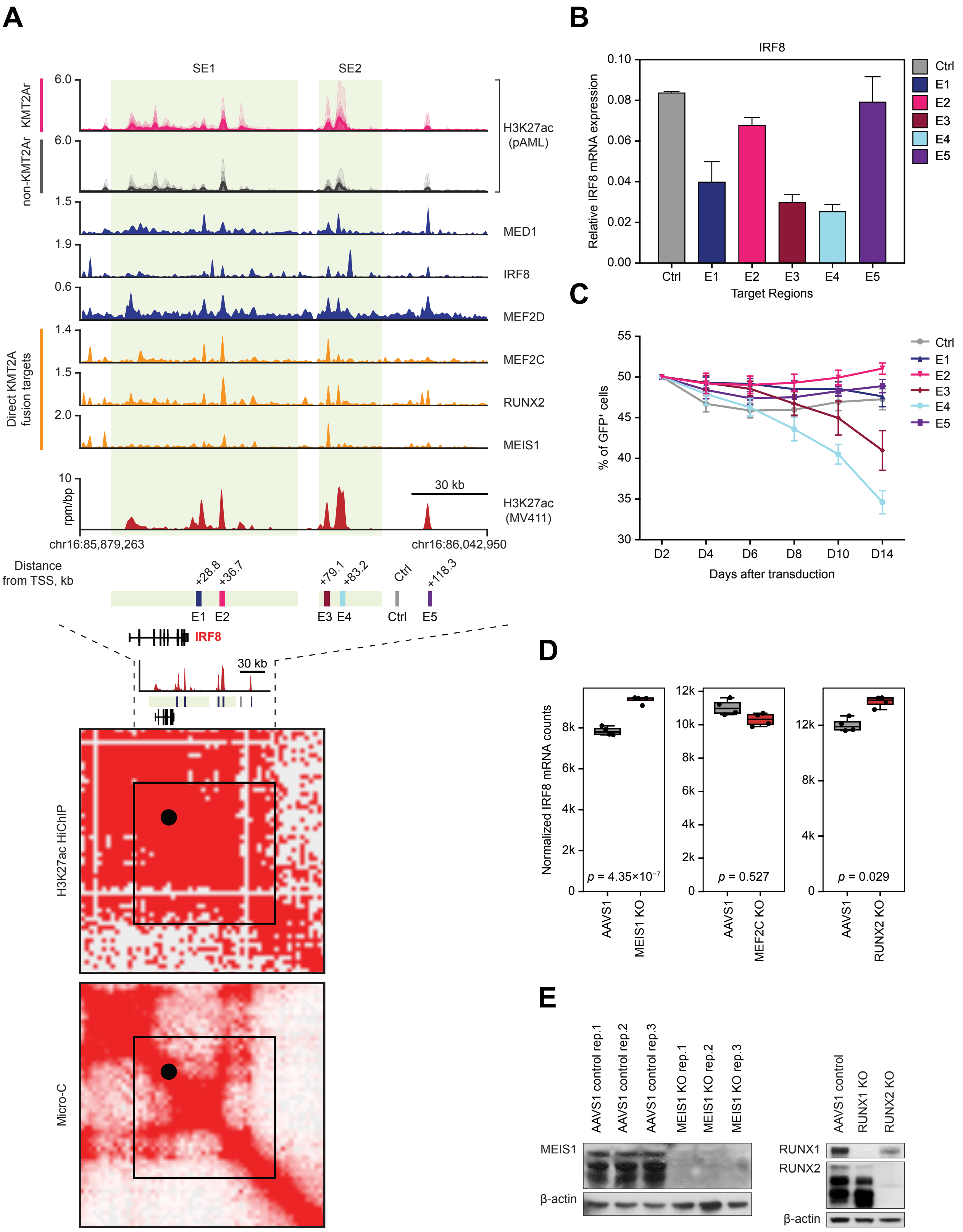
A
SE1
SE2
KMT2Ar
non-KMT2Ar
6.0
6.0
H3K27ac
(pAML)
1.5
MED1
1.9
IRF8
0.6
MEF2D
Direct KMT2A fusion targets
1.4
MEF2C
1.5
RUNX2
2.0
MEIS1
10
rpm/bp
30 kb
H3K27ac
(MV411)
chr16:85,879,263
chr16:86,042,950
Distance from TSS, kb
+28.8
+36.7
+79.1
+83.2
Ctrl
+118.3
E1
E2
E3
E4
Ctrl
E5
IRF8
30 kb
H3K27ac HiChIP
Micro-C
B
IRF8
Relative IRF8 mRNA expression
0.10
0.08
0.06
0.04
0.02
0.00
Ctrl
E1
E2
E3
E4
E5
Target Regions
C
% of GFP⁺ cells
55
50
45
40
35
30
D2
D4
D6
D8
D10
D14
Days after transduction
D
Normalized IRF8 mRNA counts
8k
6k
4k
2k
0
p = 4.35×10⁻⁷
AAVS1
MEIS1 KO
12k
10k
8k
6k
4k
2k
0
p = 0.527
AAVS1
MEF2C KO
12k
8k
4k
0
p = 0.029
AAVS1
RUNX2 KO
E
AAVS1 control rep.1
AAVS1 control rep.2
AAVS1 control rep.3
MEIS1 KO rep.1
MEIS1 KO rep.2
MEIS1 KO rep.3
MEIS1
β-actin
AAVS1 control
RUNX1 KO
RUNX2 KO
RUNX1
RUNX2
β-actin

Figure S12, related to figure 7. Structure of the IRF8 locus and mechanism of its activation in KMT2A-rearranged AML.

A, H3K27 acetylation, TF binding and 2D structure at the IRF8 locus. The top two tracks are H3K27ac metatracks representing primary AMLs. *Red*, tracks from pediatric patients with KMT2Ar AML (*n*=10); *grey*, tracks from the pediatric patients with non-KMT2Ar AML (*n*=9). Each metatrack is a collection of semi-transparent area plots representing individual samples and the average profile is represented by a thick line. The rest of the ChIP-seq tracks represent TF binding and H3K27 acetylation profiles in the MV411 cell line. Orange tracks represent TFs known to be the direct targets of KMT2A fusion oncoproteins and hypothesized to play a role in inducing IRF8 expression. Shaded areas represent 2 superenhancers associated with the IRF8 locus (SE1 and SE2). E1-E5 represent the constituent enhancers selected for the dCas9-KRAB-MeCP2 enhancer interference experiment. Superenhancer-promoter loops are demonstrated by the H3K27ac HiChIP and Micro-C 2D contact maps obtained in MV411 cells.

B, MV411 cells stably expressing dCas9-KRAB-MECP2 were transduced with lentiviral vectors encoding gRNAs targeting the E1-E5 enhancers highlighted in panel A. Expression of IRF8 was measured by RT-qPCR.

C, Viability of MV411 cells stably expressing dCas9-KRAB-MECP2 and transduced with GFP-expressing lentiviral vectors encoding gRNAs targeting the indicated enhancers was measured as fraction of GFP-positive cells.

D, Knockouts of the 3 core regulatory TFs directly activated by KMT2A fusion oncoproteins do not decrease IRF8 expression. Expression of IRF8 was measured by RNA-seq 72 hours following electroporation of MV411 cells with pre-assembled Cas9/sgRNA complexes targeting the indicated TF genes. A guide RNA targeting the AAVS1 “safe harbor” locus was used as a negative control. P-values were calculated by BH-adjusted Wald test.

E, Validation of gene knockouts by Western blotting. MV411 cells were electroporated with pre-assembled Cas9/sgRNA complexes targeting the indicated genes and protein depletion was verified by Western blot 72 hours post electroporation. Paralog-specific knockout of RUNX2 is shown. Western blot visualizing the MEF2C knockout is found in Figure 6H.

Figure S13

A

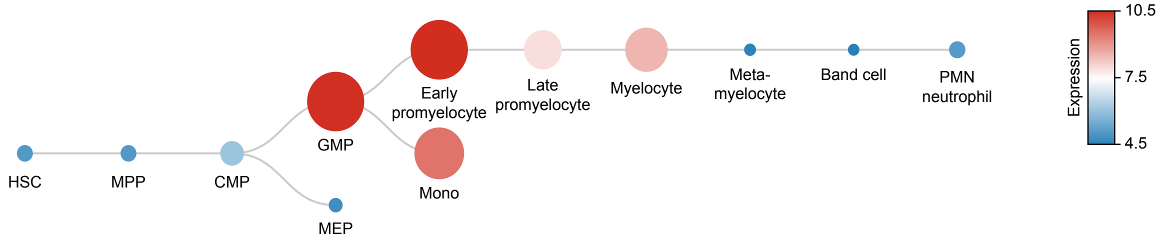

B

Differentiation of Hematopoietic cells in mRNA space
(data from Corces *et al.*, 2016)

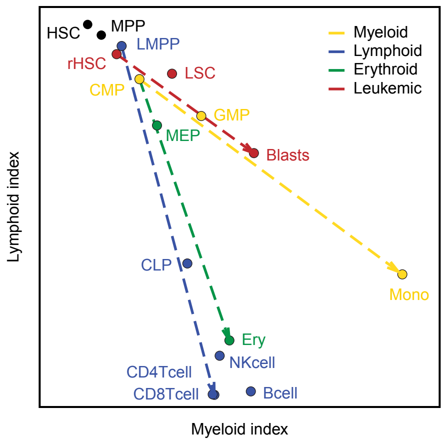

C

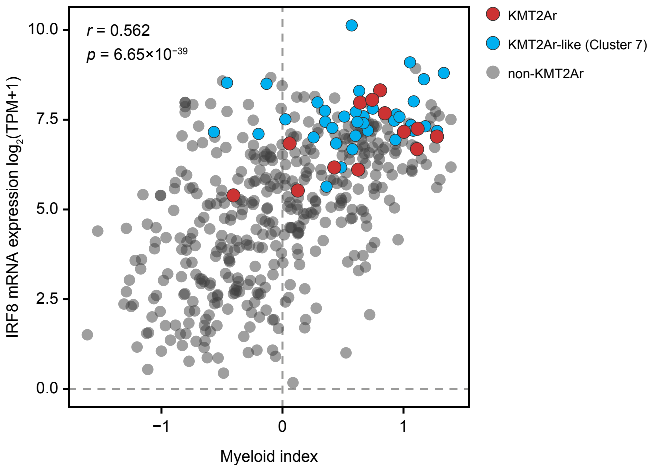

Figure S13, related to figure 7. Expression of IRF8 correlates with the myeloid differentiation state.

A, Expression of IRF8 during normal human hematopoiesis. The plot was generated using the Bloodspot database with primary data from GSE42519 (Bagger et al. 2016; Rapin et al. 2014).

B, Normal progenitors and leukemic cells are plotted according to their indices of myeloid and lymphoid development using primary data from (Corces et al. 2016). A myeloid differentiation index, computed from the mRNA expression of 19 cell type-specific markers (Methods), correctly reproduced both the normal myeloid trajectory and the functional AML hierarchy.

C, Expression of IRF8 correlates with the myeloid differentiation index in the BeatAML dataset. KMT2A-rearranged and KMT2A-like leukemia samples are color-coded as shown.

Supplementary Figure References

Bagger FO, Sasivarevic D, Sohi SH, Laursen LG, Pundhir S, Sønderby CK, Winther O, Rapin N, Porse BT. 2016. BloodSpot: a database of gene expression profiles and transcriptional programs for healthy and malignant haematopoiesis. *Nucleic Acids Res* 44: D917-24.

Corces MR, Buenrostro JD, Wu B, Greenside PG, Chan SM, Koenig JL, Snyder MP, Pritchard JK, Kundaje A, Greenleaf WJ, et al. 2016. Lineage-specific and single-cell chromatin accessibility charts human hematopoiesis and leukemia evolution. *Nat Genet* 48: 1193–1203.

Kuleshov MV, Jones MR, Rouillard AD, Fernandez NF, Duan Q, Wang Z, Koplev S, Jenkins SL, Jagodnik KM, Lachmann A, et al. 2016. Enrichr: a comprehensive gene set enrichment analysis web server 2016 update. *Nucleic acids research* 44: W90-7.

Network CGAR. 2013. Genomic and epigenomic landscapes of adult de novo acute myeloid leukemia. *New Engl J Medicine* 368: 2059–2074.

Rapin N, Bagger FO, Jendholm J, Mora-Jensen H, Krogh A, Kohlmann A, Thiede C, Borregaard N, Bullinger L, Winther O, et al. 2014. Comparing cancer vs normal gene expression profiles identifies new disease entities and common transcriptional programs in AML patients. *Blood* 123: 894–904.

Tyner JW, Tognon CE, Bottomly D, Wilmot B, Kurtz SE, Savage SL, Long N, Schultz AR, Traer E, Abel M, et al. 2018. Functional genomic landscape of acute myeloid leukaemia. *Nature* 562: 526–531.
